# Diet, Environments, and Gut Microbiota. A Preliminary Investigation in Children Living in Rural and Urban Burkina Faso and Italy

**DOI:** 10.3389/fmicb.2017.01979

**Published:** 2017-10-13

**Authors:** Carlotta De Filippo, Monica Di Paola, Matteo Ramazzotti, Davide Albanese, Giuseppe Pieraccini, Elena Banci, Franco Miglietta, Duccio Cavalieri, Paolo Lionetti

**Affiliations:** ^1^Institute of Biology and Agrarian Biotechnology, National Research Council, Pisa, Italy; ^2^Department of Neuroscience, Psychology, Drug Research and Child Health, University of Florence, Meyer Children Hospital, Florence, Italy; ^3^Department of Experimental and Clinical Biomedical Sciences, University of Florence, Florence, Italy; ^4^Fondazione E. Mach, Research and Innovation Centre, Trento, Italy; ^5^Centro di Servizi di Spettrometria di Massa, University of Florence, Florence, Italy; ^6^Institute of Biometereology, National Research Council, Florence, Italy; ^7^Department of Biology, University of Florence, Florence, Italy

**Keywords:** microbiota, diet, environment, urbanization, Africa, children, short chain fatty acids

## Abstract

Diet is one of the main factors that affects the composition of gut microbiota. When people move from a rural environment to urban areas, and experience improved socio-economic conditions, they are often exposed to a “globalized” Western type diet. Here, we present preliminary observations on the metagenomic scale of microbial changes in small groups of African children belonging to the same ethnicity and living in different environments, compared to children living on the urban area of Florence (Italy). We analyzed dietary habits and, by pyrosequencing of the 16S rRNA gene, gut microbiota profiles from fecal samples of children living in a rural village of Burkina Faso (*n* = 11), of two groups of children living in different urban settings (Nanoro town, *n* = 8; Ouagadougou, the capital city, *n* = 5) and of a group of Italian children (*n* = 13). We observed that when foods of animal origin, those rich in fat and simple sugars are introduced into a traditional African diet, composed of cereals, legumes and vegetables, the gut microbiota profiles changes. Microbiota of rural children retain a geographically unique bacterial reservoir (*Prevotella*, *Treponema*, and *Succinivibrio*), assigned to ferment fiber and polysaccharides from vegetables. Independently of geography and ethnicity, in children living in urban areas these bacterial genera were progressively outcompeted by bacteria more suited to the metabolism of animal protein, fat and sugar rich foods, similarly to Italian children, as resulted by PICRUSt (Phylogenetic Investigation of Communities by Reconstruction of Unobserved States), a predictive functional profiling of microbial communities using 16S rRNA marker gene. Consequently, we observed a progressive reduction of SCFAs measured by gas chromatography–mass spectrometry, in urban populations, especially in Italian children, respect to rural ones. Our results even if in a limited number of individuals point out that dietary habit modifications in the course of urbanization play a role in shaping gut microbiota, and that ancient microorganisms, such as fiber-degrading bacteria, are at risk of being eliminated by the fast paced globalization of foods and by the advent of westernized lifestyle.

## Introduction

Microbial colonization of the gastrointestinal (GI) tract is a fundamental process in human life cycle since microbiota–host interactions influence health and disease (Qin et al., 2010; Collado et al., 2012; Human Microbiome Project Consortium, 2012; Tremaroli and Backhed, 2012; Rodriguez et al., 2015). Animal and human hosts and their microbiota have co-evolved together over the millennia into a homeostatic and symbiotic relationship.

Dietary habits are one of the main factors contributing to the diversity of human gut microbiota (De Filippo et al., 2010; David et al., 2014). Dietary changes and gut microbiota alterations have the potential to profoundly affect host health and development (Palmer et al., 2007; Agans et al., 2011; Ringel-Kulka et al., 2013).

In infants, GI colonization is influenced by several factors including genetics, gestational age, mode of birth, diet, environment, sanitation, and antibiotic treatment (Adlerberth and Wold, 2009; Marques et al., 2010; Khodayar-Pardo et al., 2014). During the first year of life, dietary richness and environmental exposures increase and in parallel, the richness and complexity of the GI microbiota also increase (Koenig et al., 2011; Yatsunenko et al., 2012). Early life is a distinctive stage for the microbiota also in terms of functional acquisition (Yatsunenko et al., 2012). However, microbial colonization in children, following dietary and environmental changes, is still being completely uncovered.

In the last few years, researchers have gone beyond the Western world, to investigate human populations still living in rural environments and having a lifestyle completely different from that of developed countries. Metagenomic datasets obtained worldwide in different populations show that both host and environmental factors, especially diet, can affect gut microbial ecology over a lifetime (Borenstein et al., 2008; Freilich et al., 2009).

Our previous study showed for the first time that gut microbiota from children living in a rural African village in Burkina Faso, an environment resembling that of Neolithic subsistence farmers, is completely different from the microbiota of children living in the urban Western world (De Filippo et al., 2010). We demonstrated that in children different dietary habits (a fiber-rich diet of rural populations versus a typical Western diet rich in fat, animal proteins, and simple sugars) affect the gut microbiota.

A fiber and plant-derived polysaccharide-rich diet in children and adults is associated with a human gut microbiota enriched in Bacteroidetes phylum compared to Firmicutes (De Filippo et al., 2010; Yatsunenko et al., 2012; David et al., 2014). The fermentation of non-digestible carbohydrates stimulates the growth of bacterial producers of short-chain fatty acids (SCFAs) that are associated with disease and health in different ways (Tan et al., 2014).

Since the publication of our study, the impact of diet on gut microbiota has been observed and reported in different geographically isolated populations, such as Amazonas from Venezuela and rural populations in Malawi (Yatsunenko et al., 2012) and Papua New Guinea (Martinez et al., 2015). In addition, there are reports about drastic and rapid changes in gut microbiota when adults make a dietary switch from carnivorous to vegetarian diets (David et al., 2014; Gomez et al., 2016). The study of gut microbiota in ancestral populations, such as Hazda hunter-gatherers from Tanzania, one of the last few remaining populations with a Paleolithic type diet and lifestyle (Schnorr et al., 2014), showed significant differences in microbiota due to dietary fluctuations linked to seasonal changes. The microbiota of BaAka hunter-gatherers and Bantu agriculturalists is characterized by microbial gradients linked to traditional subsistence strategies (Gomez et al., 2016).

Altogether, the studies on traditional and culturally diverse populations living in isolation from the globalized world showed the degree of an individual’s traditional lifestyle and can explain the evolutionary processes of human gut microbiota.

We hypothesized that in the same country the phenomenon of urbanization, and consequently the “Westernization” may affect dietary habits of traditional populations, resulting in modification of gut microbiota profiles. The aim of the present preliminary investigation was to assess the impact of dietary habits on the gut microbiota of small groups of African children who live in a rural village, with marginal contacts with the globalized world, respect to children living in suburban small town and in an urban area. For such populations who live in the same country and belong to the same ethnic group, such a transition is in parallel with increased wealth and greater food availability.

We integrated the data on the gut microbiota characterization of our previous study on children living in the rural villages of Boulpon (district of Nanoro) in Burkina Faso (De Filippo et al., 2010), with data on microbiota from children of the same Mossi ethnicity who live in the small town of Nanoro and from those of wealthy families living in Ouagadougou, the capital city. We then compared the composition of the gut microbiota of these three African populations, corresponding to different levels of urbanization, with that of previously known Italian children, as representative of a typical Western and urbanized population.

## Materials and Methods

### Enrollment of Children Populations and Fecal Sample Collection

In this study, we enrolled 11 healthy children living in the rural village of Boulpon (Boulkiemde province, Burkina Faso, BR), 8 healthy children living in the small town of Nanoro (Boulkiemde province, Burkina Faso, BT), 5 children living in the capital city of Burkina Faso, and 13 healthy children living in the urban area of Florence, Italy (EU). All children aged 2–8 years, had not taken antibiotics or probiotics in the 6 months prior to the sampling dates and had not been hospitalized in the previous 6 months. A detailed medical and lifestyle report was obtained from EU children’s parents as well as a 4-day dietary questionnaire and an in-depth interview on African children’s diet was obtained directly from their mothers. Additional data were collected for all children (Supplementary Table 1) including ethnicity, environment in which they live, and mode of birth (natural or cesarean). All children were breastfeed as infants, except one EU children (1EU) who was formula-fed.

Despite the high incidence of infectious disease, including malaria and malnutrition in the area, all children were healthy at the time of sample collection. BC children were healthy and belonging to wealthy families. For BR and BT children, upper mid-arm measurement excluded both severe and moderate malnutrition. As representative of a healthy Western population, we selected children of the same age who are generally concordant for growth, socially homogeneous and eating the diet and living in an environment typical of the developed and urbanized world. This study was carried out in accordance with the recommendations of the Ethical Committee of Meyer Children Hospital, Florence – Italy. All children’s parents were made aware of the nature of the experiment and gave written informed consent in accordance with the sampling protocol approved by the Ethical Committee of Meyer Children Hospital, Florence, Italy and in accordance with the Declaration of Helsinki.

Fecal samples of African and Italian children were collected in the morning, 1–2 h after the first meal, by physicians or parents and preserved in RNAlater (Qiagen) at -80°C until extraction of genomic DNA. All samples were processed in the same way as reported in the successive paragraph in the laboratory of University of Florence (Supplementary Materials).

### Bacterial Genomic DNA Extraction from Fecal Samples

The bacterial genomic DNA was extracted as previously reported (De Filippo et al., 2010). The procedure was based on a modified protocol (Supplementary Materials) proposed by Zoetendal et al. (2006).

### Pyrosequencing

For each sample, we amplified the 16S rRNA gene using the special fusion primer set specific for V5–V6 hypervariable regions and corresponding to primers 784F and 1061R described by Andersson et al. (2008), and using the FastStart High Fidelity PCR system (Roche Life Science, Milano, Italy). The 454 pyrosequencing was conducted outsourcing by DNAVision Agrifood S.A. (Liège, Belgium) on the GS FLX+ system using the Titanium chemistry following the manufacturer recommendations (Supplementary Materials).

### Data Analysis

Sequence data of BT and BC samples are available at http://www.ebi.ac.uk/ena/data/view/PRJEB19895, under the accession number PRJEB19895. Data of BR and EU samples are available at http://www.ebi.ac.uk/ena/data/view/ERP000133, under the accession number ERP000133, as previously reported (De Filippo et al., 2010). Raw 454 files were demultiplexed using Roche’s sff file software. Reads of all data sets were pre-processed altogether using the MICCA pipeline (v. 1.5) (Albanese et al., 2015). *De novo* sequence clustering, chimera filtering and taxonomy assignment were performed by micca-otu-de novo (parameters -s 0.97 -c), after trimming and quality filtering (Supplementary Materials).

Operational taxonomic units (OTUs) were assigned by clustering the sequences with a threshold of 97% pair-wise identity, and their representative sequences were classified using the RDP software version 2.7 (Wang et al., 2007). Template-guided multiple sequence alignment was performed using PyNAST57 (v. 0.1) (Caporaso et al., 2010) against the multiple alignment of the Greengenes 16S rRNA gene database (DeSantis et al., 2006) filtered at 97% similarity. Sampling heterogeneity was reduced by rarefaction, obtaining 12,964 sequences per sample.

Alpha (Chao1 index and Shannon entropy) and beta diversity [UniFrac-(Lozupone et al., 2011) and Bray–Curtis dissimilarities] were calculated using the Phyloseq package (McMurdie and Holmes, 2014) of the R software suite. Principal coordinates analysis (PCoA) using the phyloseq package of the R software suite was performed. The significance of between-groups differentiation on the UniFrac distances and Bray–Curtis dissimilarity was assessed by PERMANOVA using the adonis() function of the R package vegan with 999 permutations.

To compare the relative abundances of OTUs among the four groups, the two-sided, unpaired Wilcoxon test was computed, removing taxa not having a relative abundance of at least 0.1%, in at least 20% of the samples, and using the function mt() in the phyloseq library and the *p*-values were adjusted for multiple comparison controlling the family-wise Type I error rate (minP procedure).

Heatmap plots of percentage abundances at phylum level were obtained by using STAMP (Parks et al., 2014), and supported by dendogram, obtained with Average Neighbor and Unweighted Pair Group Method with Arithmetic Mean (UPGMA).

Bacterial species were assigned, based on Basic Local Alignment Search Tool nucleotide (BLASTn) software in the National Center for Biotechnology Information (NCBI) database, considering the highest percentage of identity (Query cover 100–99% and Identity 99 or 95%). Expectation value (E-value) was used to select significant BLAST hits, keeping only outcomes with the lowest E-value (minimal E-value of 10^-3^). To infer the functional contribution of microbial communities on 16S rDNA sequencing data set, we applied PICRUSt (Phylogenetic Investigation of Communities by Reconstruction of Unobserved States; Langille et al., 2013). The functional pathways discovery and related statistical significance were assessed by using the linear discriminant analysis (LDA) effect size (LEfSe) method (Segata et al., 2011). An alpha significance level of 0.05, either for the factorial Kruskal–Wallis test among classes or for the pairwise Wilcoxon test between subclasses, was used. A size-effect threshold of 2.0 on the logarithmic LDA score was used.

### Determination of Short-Chain Fatty Acids (SCFAs) in Fecal Samples

Concentrations of fecal SCFAs were determined from 250 mg frozen fecal samples, according to the previous protocol (De Filippo et al., 2010), by solid-phase microextraction- gas chromatography-mass spectrometry (SPME-GC-MS) using a Varian Saturn 2000 GC–MS instrument with 8200 CX SPME autosampler. The SCFAs concentration in fecal sample was expressed in μmol/g of feces. To determine statistical significance of differences observed among the four populations we used unpaired Student’s *t*-test (one tailed).

## Results and Discussion

### Change of Dietary Habits in Burkina Faso Children Living in Rural and Urban Areas

In order to investigate the effect of diet modifications, corresponding to different socio-economic conditions and food availability, on gut microbial communities, we studied dietary habits of three populations of healthy children, living in different areas of Burkina Faso. All children belong to the Mossi ethnic group (the largest in Burkina Faso comprising 74.9% of the Burkinabè population). The age range of all groups of children was 2–8 years, with an average of 4.6 ± 1.5 years (mean ± SE). Age and gender characteristics of each group are reported in Supplementary Table 1.

**FIGURE 1:**
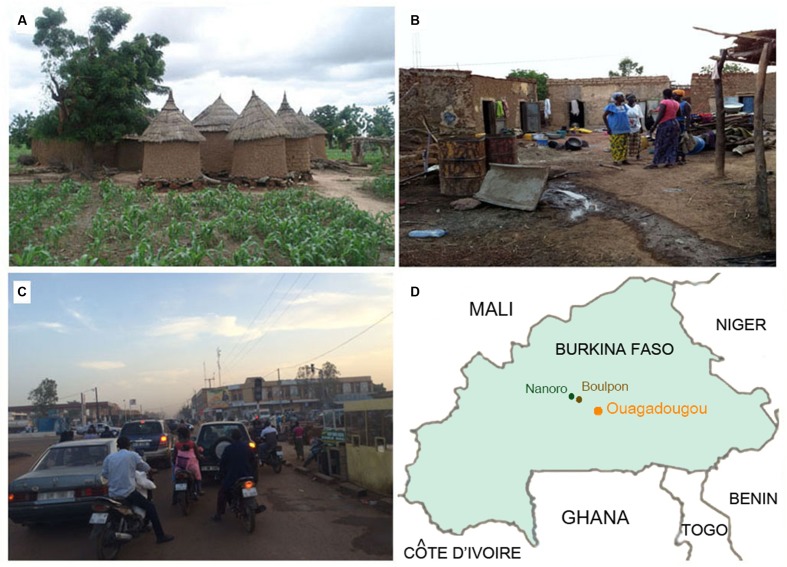
Rural and urban environments in Burkina Faso. **(A)** Rural village in Boulpon, **(B)** urban village in Nanoro town, **(C)** Ouagadougou, the capital city of Burkina Faso (personal photographs of Prof. P. Lionetti), and **(D)** map of Burkina Faso.

We compared a previously analyzed population (De Filippo et al., 2010) of 11 children (rural, BR) living in Boulpon, a typical rural village of Burkina Faso (Boulkiemdé province, Nanoro department; geographic coordinates 12°39′N 2°4′W) with 8 children (town, BT) living in Nanoro (geographic coordinates 12°41′N 2°12′W), a small African town surrounded by rural villages, corresponding to an initial urbanization status, and 5 children (Capital city, BC) from wealthy families, living in the capital city of Burkina Faso, Ouagadougou (geographic coordinates 12°21′26″N 1°32′7″W; **Figure 1**), about 90 Km from Nanoro (**Figure 1**).

The Boulpon village consists of a cluster of huts built using wood and straw (**Figure 1A**), in which the population live in communities based on subsistence agriculture. Nanoro is a small town with about 5,200 inhabitants, consisting of urban agglomerates of small brick houses (**Figure 1B**). Ouagadougou is the capital city of Burkina Faso, with a population of 1,475,223 inhabitants and is the administrative, cultural, economic and industrial center of the nation (**Figure 1C**). The industry of Ouagadougou is the sector that fuels urban growth, as people move to the city from the countryside to find employment in processing plants and factories.

All study children were healthy at the time of the investigation. However, BR children were poor, at high risk of infectious diseases and malnutrition, while BT children lived in average economic conditions for Burkina Faso which meant a dirty environment (**Figure 1B**), and were at risk of infectious disease but at low risk of malnutrition. The BC children were from wealthy families living in clean, modern houses, at no risk of malnutrition and at lower risk of infectious diseases when compared to the other groups of African children, but certainly at higher risk of infections when compared to Italian children. It is worth of mentioning that the capital city of Ouagadougou is very polluted by vehicle exhaust, especially during the dry season.

The dietary habits of these three African populations were compared to a population of our previous study comprised of 13 European children (EU) living in Florence (Italy) and having a typical Western diet (De Filippo et al., 2010).

Initially, we analyzed the dietary habits and daily food intake of all our pediatric populations, based on dietary questionnaires and interviews with mothers and care givers, estimating average quantities of food ingested per day, food energy (kcal/day), grams of protein, fat, carbohydrates, including simple sugars, and fiber (Supplementary Table 2).

As shown in our previous study (De Filippo et al., 2010), the diet of BR children is predominantly vegetarian, rich in fibers and plant-polysaccharides and low in fat, animal protein, and simple sugars (Supplementary Table 2A). The sources of fibers are also quite unique, as derived from locally cultivated indigenous cereals (millet, *Panicum miliaceum* and sorghum, *Sorghum vulgare*), legumes (Niebè, *Vigna unguiculata*), vegetables (Néré, *Parkia biglobosa* and baobab leaves), fruits (especially mango, papaya, and bananas), and fermented products (Soumbalà from Nerè seeds; Supplementary Figure 1). Millet and sorghum, typical cereals of the Burkinabè diet in these rural villages, are frequently still ground into flour on a flat grinding stone, similarly to what humans did during the agriculture revolution in the Neolithic age, to produce a thick porridge called Tô, that is the principal dish-component of Burkinabè meals.

The BT children living in Nanoro town still eat cereals and legumes, similarly to BR children but, depending on their socio-economic status, their diet also contains rice, corn, peanuts, peanut oil, and about once-a-week mutton or chicken from animals bred in the village or dried fish. Unlike the BR population, cereal flour, legumes, fruit, and dried fish can be bought in the local market in Nanoro town, where products from neighboring countries can also be found (Supplementary Table 2B).

The BC children, living in the capital city of Ouagadougou, eat a typical African diet of cereals (millet, sorghum, rice, soya) and legumes (Niebè), but also bread, milk and dairy products such as cheese and yogurt, eggs, fruit juices, snacks, sweet bakery products and different kinds of meat and fish, including frozen fish no more than three times *per* week. Their diet, therefore, is very similar to that of children living in an industrialized and globalized world. Many of these products are bought at supermarkets (Supplementary Table 2C).

The Italian children in our study eat a typical Western diet, high in starch, simple sugars, animal protein, and fat and low in fiber (Supplementary Table 2D), as previously described (De Filippo et al., 2010).

**FIGURE 2:**
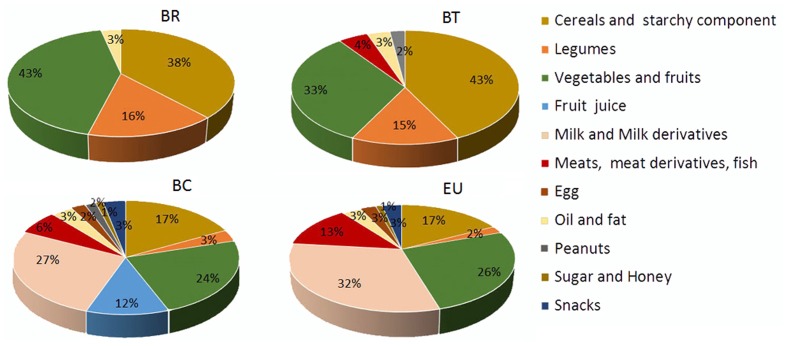
Variety of food consumption in the four children populations. Pie charts indicate the percentages of daily foods assumed by the African and European populations. BR: children form rural village; BT: children from Nanoro town; BC: children from capital city of Burkina Faso; EU: children from Europe (Florence, Italy).

Our nutritional analysis of the four populations of children showed that passing from rural (BR) to urban centers (BT, BC, and EU), the variety of food consumed increased (**Figure 2**), as well as the daily intake of fat, protein and simple sugars, and consequently the daily caloric intake, whereas the fiber intake was progressively reduced (Supplementary Table 2). The average amount of fiber in the BR diet is 14.2 g/day (3.19% of total grams of daily food intake) compared with 12.5 g/day (2.72%) in the BT diet, 9.7 g/day (1.04%) in the BC, and 8.4 g/day (0.9%) in the EU diet. Fiber intake diminished as the average daily calorie intake increased (BR: 996 kcal/day; BT: 1094.5 kcal/day; BC: 1454.3 kcal/day; EU: 1512.7 kcal/day; Supplementary Table 2).

Despite the limitation of the small sample number, our groups of African children clearly reflect different dietary habits in populations of the same ethnic group and belonging to the same geographic area, but living either in rural or urban areas, and having different socio-economic conditions.

### Fiber Intake Is Correlated with Levels of Fecal Short Chain Fatty Acids

**FIGURE 3:**
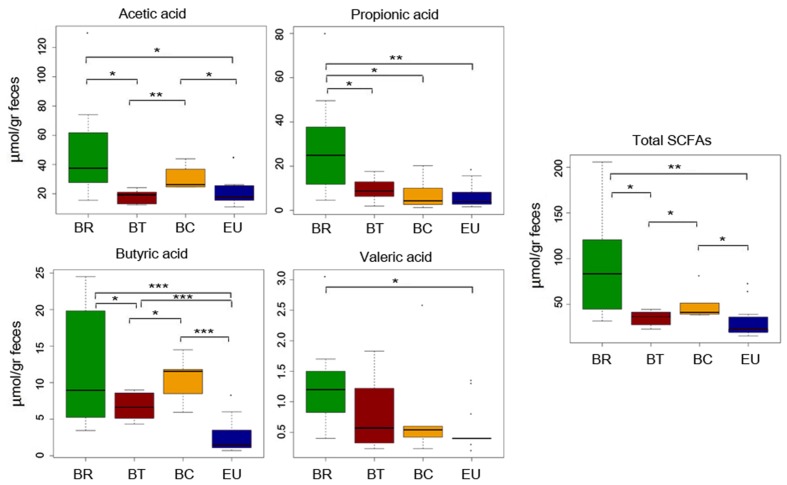
Quantification of SCFAs levels in fecal samples from African and EU populations by SPME-GC-MS. Mean values ( ± SEM) are plotted. Comparison among groups by one-tailed Student’s *t*-test. ^∗^*p* < 0.05; ^∗∗^*p* ≤ 0.01; ^∗∗∗^*p* ≤ 0.001.

In our previous study (De Filippo et al., 2010) comparing dietary habits of BR and EU populations, we observed a correlation between fiber intake and fecal levels of SCFAs. Thus, considering the differences in food consumption, especially in fiber intake, in BT and BC populations compared with BR, in the present study we analyzed metabolomics by SPME-GC-MS, to measure SCFA levels (Supplementary Table 3). We observed a significant reduction in total fecal SCFAs in children who live in urban centers with respect to BR children (**Figure 3**; *p*-values by one-tailed Student’s *t*-test; Supplementary Table 4). We found a clear trend, especially for propionic and valeric acids (**Figure 3**; *p*-values by one-tailed Student’s *t*-test; Supplementary Table 4). Fecal samples of BC showed a significant increase in total SCFAs, especially for acetic and butyric acids compared to BT and EU, but a decrease compared to BR (**Figure 3**; *p*-values by one-tailed Student’s *t*-test; Supplementary Table 4). Interestingly, the EU children’s fecal samples contained significantly low levels of SCFAs, especially butyric acid, compared to the three African groups (**Figure 3**; *p*-values by one-tailed Student’s *t*-test; Supplementary Table 4).

### Microbiota Characterization of Different Populations of African Children Compared to Europeans: Taxonomic Changes as an Effect of Diet and Environment

We compared the meta-taxonomic data of BT and BC populations, obtained by pyrosequencing (454 FLX, Roche) of the V5–V6 hypervariable regions of the 16S rRNA gene with the previous results obtained with the same methodologies for BR and EU populations (De Filippo et al., 2010).

We evaluated microbial richness (alpha diversity) among populations. Observed OTUs and the Chao1 index indicated a downward trend (although not significant according to PERMANOVA analysis) in species richness from rural BR to BC and EU children, whereas BT children showed a high alpha diversity index. The Shannon index, estimating entropy, indicated reduced alpha diversity in BR children compared to the other populations (Supplementary Figure 2).

**FIGURE 4:**
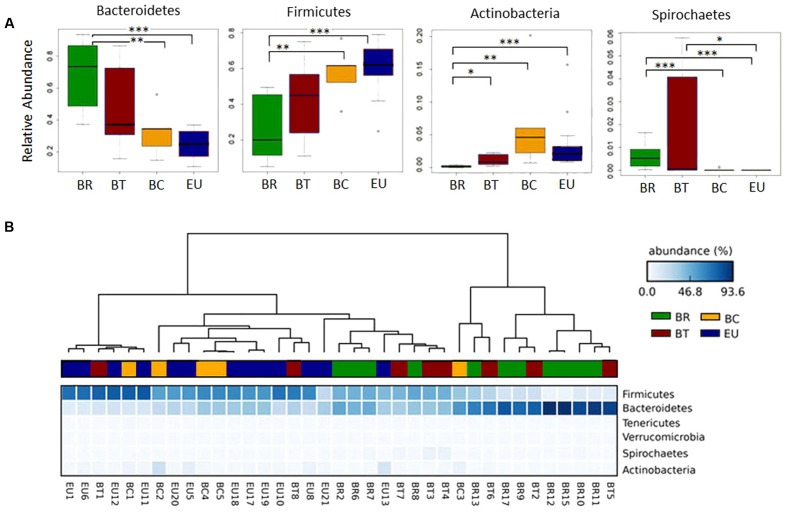
Clustering of African and European populations based on microbiota composition. **(A)** Box plot of relative abundances of the statistically significant different major phyla in African and European populations (Wilcoxon rank sum test; ^∗^*p*-value < 0.05, ^∗∗^*p*-value < 0.01, ^∗∗∗^*p*-value < 0.001). **(B)** Heatmap plot indicating the sequence abundances (percentage; blue-scale squares) assigned at each phylum in each sample. Dendrograms, obtained with Average Neighbor UPGMA method, are used to cluster each fecal sample of the children populations (horizontal) based on phyla abundances. Each sample, belonging to respective group, is represented by a different color: green = BR, brown = BT, yellow = BC, and blue = EU.

At phylum level, the rural populations had a higher ratio of Bacteroidetes to Firmicutes, but this ratio gradually diminished in fecal samples from children in more urban settings (**Figure 4**). Bacteroidetes were abundant especially in BR and BT children (68.6 and 47.7% respectively) compared to 32.6% in BC and 25.9% in EU children with a significantly higher abundance when comparing BR with BC and EU (**Figure 4A**; Wilcoxon rank-sum test; Supplementary Table 5A). Conversely, Firmicutes were more abundant in BC and EU children (57.5 and 60.2%, respectively) than in BT, and significantly higher than in BR children (**Figure 4A**; Wilcoxon rank-sum test; Supplementary Table 5A).

Actinobacteria were more abundant in BC compared to the other African and European populations (6.74% in BC vs. 3.6% in EU, 0.17% in BR, and 1.11% in BT), and significantly reduced in BR compared to the other groups (**Figure 4A**; Wilcoxon rank-sum test; Supplementary Table 5A). An abundance of Proteobacteria was observed in BT and EU (3.8% in BT and 4.94% EU vs. 2.73% in BR and 1.26% in BC; Supplementary Figure 3), compared with other populations, although not statistically significant.

Among the minor phyla of gut microbiota, Spirochaetes was significantly increased in BR compared with BC and EU children, as well as in BT compared to EU children (**Figure 4A**; Wilcoxon rank-sum test). Tenericutes and Verrucomicrobia were relatively increased in the BT population compared with the other groups (Supplementary Figure 3).

BT populations showed a greater variability in the relative abundance of phyla (**Figure 4A**) compared with BC and EU children, suggesting an inter-individual variability in microbiota composition. One possible explanation for this phenomenon could be the increased variety of food consumption with the addition of dietary products of animal origin to a typical rural Africa vegetarian diet.

Based on sequence abundances at phylum level in microbiota of each population, the dendogram, obtained with the Average Neighbor and Unweighted Pair Group Method with Arithmetic Mean (UPGMA), showed a clear separation between BR and EU populations (**Figure 4B**), especially due to the different ratio Bacteroidetes/Firmicutes, as previously observed (De Filippo et al., 2010). In contrast, BT and BC children showed a progressive shift toward the phyla distribution observed in EU children in accordance with their different dietary habits and environments.

Whereas most BC children clustered together with EU, two out of eight BT samples clustered closely to the EU group, due to the relative abundance of Firmicutes. We think this is due to the fact that BT children living in Nanoro did not have a uniform diet, with two of them having a more Westernized diet and the others eating very similarly to the rural area diet, as revealed in the questionnaire we administered. However, the majority of BT children fell within the BR cluster, or in a sub-cluster interposed between BR and EU population, due to the abundance in Bacteroidetes (**Figure 4B**).

Although the limited number of samples have to be considered, these results, confirmed also by dendograms obtained at family and genus levels (Supplementary Figure 4), suggest that Burkina Faso children of the Mossi ethnic group who live in the same geographic area have different gut microbiota composition, according to the ways in which their diets and the environment where they live, have changed.

Following the taxonomic assignment at family level, we observed that *Prevotellaceae*, the most abundant family in BR and BT populations (66.8 and 41.14% mean relative abundance respectively), were significantly more abundant in rural BR children compared with BC and EU with a clear reduction in BC (10.4%), and almost absent in EU children (0.44%) (Supplementary Figure 5; Wilcoxon rank-sum test; Supplementary Table 5B). Conversely, several bacterial families were less abundant in the BR population and progressively more present in urban African (BT and BC) and EU children. Among these, *Bacteroidaceae, Bifidobacteriaceae, Porphyromonadaceae*, and *Rikenellaceae* were significantly decreased in BR compared with BC and EU, and in BT compared with EU children, as were *Lachnospiraceae* and *Ruminococcaceae* significantly reduced in BR compared with BC and EU children (Supplementary Figure 5; Wilcoxon rank-sum test; Supplementary Table 5B).

We also observed a significant increase in *Enterobacteriaceae* in the BT population compared with EU, and in EU compared to BR (Supplementary Figure 5; Wilcoxon rank-sum test; Supplementary Table 5B). *Spirochaetaceae* was more variable within the BT population and more abundant in the BR population compared to BC and EU (Supplementary Figure 5; Wilcoxon rank-sum test; Supplementary Table 5B). *Desulfovibrionaceae* and *Sutterellaceae* were progressively and significantly more abundant in BT, BC and EU children, and almost absent in the BR population (Supplementary Figure 5; Wilcoxon rank-sum test; Supplementary Table 5B).

**FIGURE 5:**
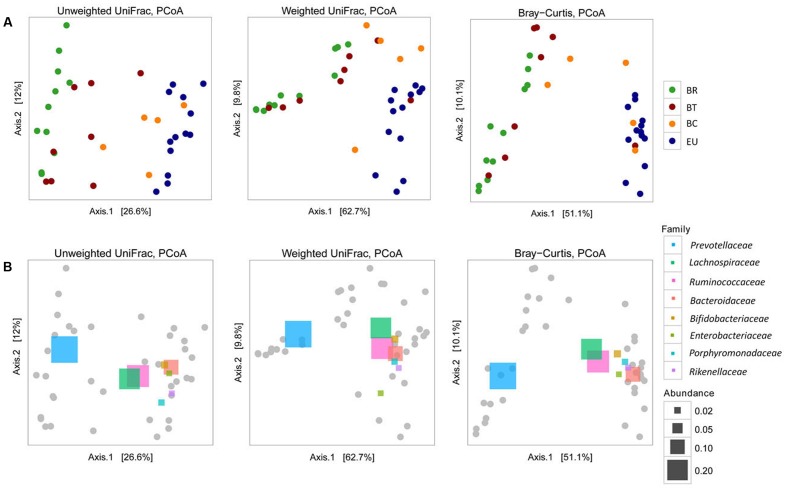
Beta diversity **(A)** Principal coordinates analysis (PCoA) derived from unweighted and weighted UniFrac and Bray–Curtis distances among samples of the four populations (*p* = 0.0001 by PERMANOVA). Colored dots representative of the four populations are as in **Figure 4**. For each axis, in square brackets, the percent of variation explained was reported. **(B)** Beta diversity and correlation with the principal abundant bacterial families, represented by colored rectangles (*p* = 0.0001 by PERMANOVA). Different sizes of rectangles indicate the size of relative family abundance. For each axis, in square brackets, the percent of variation explained was reported.

The observed distribution of the four populations based on taxonomic assignment at phylum level (**Figure 4B**) was confirmed by analysis of microbial community structure (beta diversity). Considering Unweighted and Weighted UniFrac distances and Bray–Curtis dissimilarities, PCoA analysis (**Figure 5A**) showed a clear difference between BR and EU samples, confirming the different gut microbiota composition between African and European populations (PERMANOVA analysis; unweighted-UniFrac, *p* = 0.0001; weighted-UniFrac *p* = 0.0001; Bray–Curtis *p* = 0.0001). The BT sample distribution was close to the BR population, while BC was similar to the EU (**Figure 5A**), suggesting a progressive change in gut microbial communities in African populations, as an effect of transition from the rural to the urban environment, improvement in socio-economic status and modification of dietary habits.

The different abundances of some bacterial families allow a clear discrimination of the microbiota profiles in the four populations, as highlighted by PCoA analysis based on UniFrac distances and Bray–Curtis dissimilarities (**Figure 5B**). The abundance of *Prevotellaceae* in BR and BT populations and of *Bacteroidaceae, Lachnospiraceae, Rikenellaceae, Porphyromonadaceae*, and *Enterobacteriaceae* in the EU children illustrates the variety between African and European samples. The abundance of *Lachnospiraceae* and *Ruminococcaceae* indicates how BC samples come close to EU microbiota.

### Urbanization and Improved Socio-economic Conditions Led to the Loss of Microbial Profiles Typical of Rural Populations

**FIGURE 6:**
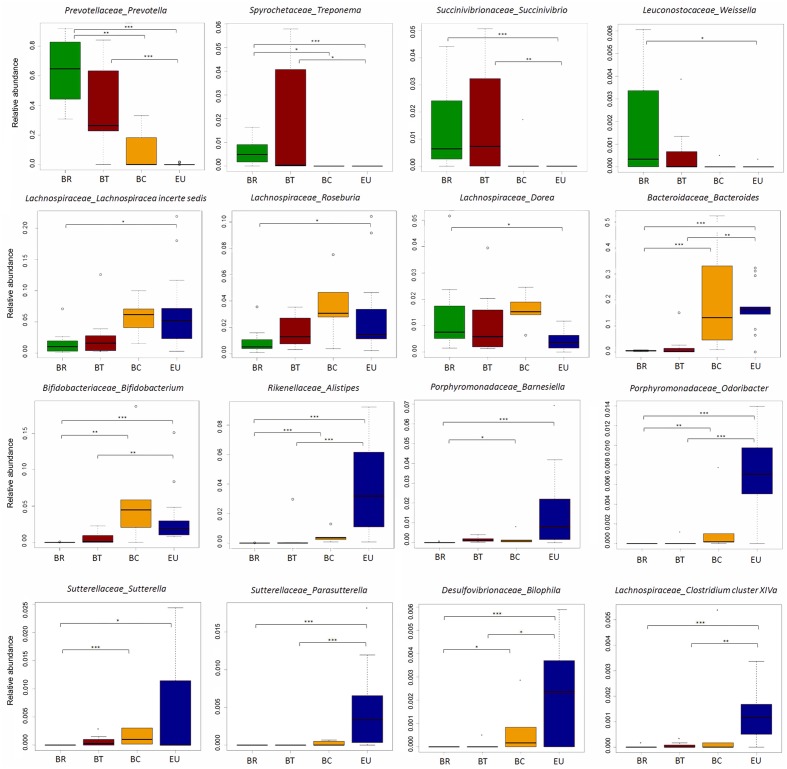
Disappearance of ancient microbial pattern and acquisition of “Western” microbial profiles passing from rural to urban environments in Burkina Faso populations. Box plot of relative abundances of the statistically significant different genera in African groups in comparison with European population (Wilcoxon pairwise test; ^∗^*p*-value < 0.05, ^∗∗^*p*-value < 0.01, ^∗∗∗^*p*-value < 0.001).

According to our previous study, at genus level, the gut microbiota of BR children was almost entirely populated by *Prevotella* (64.4% average out of total sequence amount). BT and BC populations presented a progressive decrease in its abundance compared to BR (**Figure 6**; 38.8% in BT and 10.3% in BC; Wilcoxon rank-sum test; Supplementary Table 5C), reflecting the reduction of dietary fiber intake and consequently SCFAs levels in fecal samples. It is worth noting that this genus was almost absent in EU children’s gut microbiota.

Sequence alignments by BLASTn indicated that *Prevotella* sequences were attributable with 99% of identity (see section “Materials and Methods”) to *P. copri*, *P. stercorea* and *P. melaninogenica*, over uncultured *Prevotella* spp. In our previous study, we found a set of sequences classified as *Xylanibacter*. Recently, *Xylanibacter* 16S rDNA was re-classified within the larger *Prevotella* genus (Sakamoto and Ohkuma, 2012).

Interestingly, an increase in *Treponema*, *Succinivibrio*, and *Weissella* distinguished both BR and BT from BC and EU populations (**Figure 6**; Wilcoxon rank-sum test; Supplementary Table 5C). BLASTn alignment showed that *Treponema* spp. sequences were mainly attributable with 99% of identity to *T. succinifaciens*, a known carbohydrate metabolizer isolated from the gut of termites and swine, and observed in other traditional human populations (Obregon-Tito et al., 2015). Interestingly, rural Burkina Faso populations occasionally eat cooked termites, and prepare foods on insect-contaminated surfaces, thus supporting the idea that termites and the fiber degrading microbes they harbor are likely to colonize the human gut.

*Succinivibrio* is generally associated with bovine rumen (Bryant, 1970), and was also found in higher frequency in the Hadza hunter gatherers and traditional Peruvian populations (Schnorr et al., 2014; Obregon-Tito et al., 2015) and might be involved in starch, hemicellulose, and xylan degradation, similarly to *Prevotella* and *Treponema*.

These observations confirm that the abundance of bacteria metabolizers of plant-polysaccharides, hemicellulose and xylan (De Filippo et al., 2010), are derived from high-fiber diets similarly to what is reported in children and adults from Malawi and Venezuela whose diets are dominated by plant-derived polysaccharide foods (Yatsunenko et al., 2012). When individuals do not live any more in rural areas but in urban environments, these genera decrease dramatically, to the point of being depleted in EU microbiota. Interestingly, the progressive loss of these bacteria reflects the gradual reduction in fecal SCFAs levels from BR to BT, BC, and EU populations.

Conversely, *Lachnospiraceae incertae sedis*, *Roseburia*, and *Dorea* were reduced in BR and BT children, but increased in BC, and variously represented in EU (**Figure 6**; Wilcoxon rank-sum test; Supplementary Table 5C). Thus, urbanization and a Westernized diet led to the loss of ancient microbial profiles typical of traditional and rural populations in BC children, such as *Prevotella*, and to the increase in bacterial genera associated with a Western-like diet (Wu et al., 2011; David et al., 2014; Martinez et al., 2015).

*Bacteroides* and *Bifidobacterium*, were more abundant in BC and EU children compared with BR and BT children (**Figure 6**; Wilcoxon rank sum test; Supplementary Table 5C). Enrichment of *Bacteroides* was related to lipids, cholesterol and amino acids intake, and dairy consumption, as previously observed (Wu et al., 2011; Martinez et al., 2015). *Bifidobacterium*, generally associated to infant microbiota and with milk and milk-derived food consumption, is probably related to their increased consumption of milk and dairy products respect to BR and BT children.

Finally, *Alistipes*, and *Barnesiella* distinguished Europeans from Africans (**Figure 6**; Wilcoxon rank sum test; Supplementary Table 5C), in accordance with a previous study showing enrichment of these bacterial genera in Western populations (Martinez et al., 2015).

Among the minor genera, we observed an increase in *Bilophila, Sutterella, Parasutterella, Odoribacter*, and *Clostridium cluster XIVa* (that includes *Clostridium* spp., *Eubacterium, Ruminococcus, Coprococcus, Dorea, Lachnospira, Roseburia*, and *Butyrivibrio*), in both BC and EU children (**Figure 6**; Wilcoxon rank sum test; Supplementary Table 5C).

Our results suggest that diets rich in protein and fat and poor in fiber influence the gut microbiota, independently of geographic origin. *Alistipes* and *Bilophila* have been previously linked to an animal protein-rich diet (Wu et al., 2011; David et al., 2014; Martinez et al., 2015). It has been reported that high-fat diets induce an increase in the abundance of *Bilophila wadsworthia*, a member of the *Desulfovibrionaceae* family, which generates hydrogen sulfide through taurine metabolism. An abundance of *Bilophila* and its metabolism has been associated with inflammation, as recently observed in a mouse model (Devkota et al., 2012). Members of *Sutterella* genus are known to be resistant to bile acids, and their role as commensals in human GI tract or their association with dysbiosis in some human diseases, such as Inflammatory Bowel Disease, autism, arthritis and celiac disease, remains partly controversial (Mukhopadhya et al., 2011; Williams et al., 2012; Cheng et al., 2013; Hansen et al., 2013; Wang et al., 2013; Lavelle et al., 2015; Di Paola et al., 2016).

*Clostridium XIVa* and *Odoribacter*, well-known butyrate-producer bacteria (Morgan et al., 2012; Lopetuso et al., 2013; Van den Abbeele et al., 2013), contribute to SCFAs production in BC and EU children. However, their least abundance in the gut microbiota in comparison with *Prevotella* enrichment in microbiota of BR could explain the differences in SCFAs levels found in fecal samples of rural and urban populations.

### Functional Metabolic Profiles of Gut Microbiota from African and European Children Reflect Different Dietary Habits and Environments

To evaluate how the observed taxonomic differences between the gut microbiota of African and European children affect their metabolic potential, we applied PICRUSt (Phylogenetic Investigation of Communities by Reconstruction of Unobserved States) (Langille et al., 2013), a computational approach useful for inferring the functional contribution of microbial communities on the 16S rDNA sequencing dataset. Despite the limitation of the functional inference approach, we evaluated accuracy of PICRUSt, by using the Nearest Sequenced Taxon Index (NSTI), developed to quantify the availability of nearby genome representatives for each microbiome sample (Supplementary Materials and Methods and Table 6).

**FIGURE 7:**
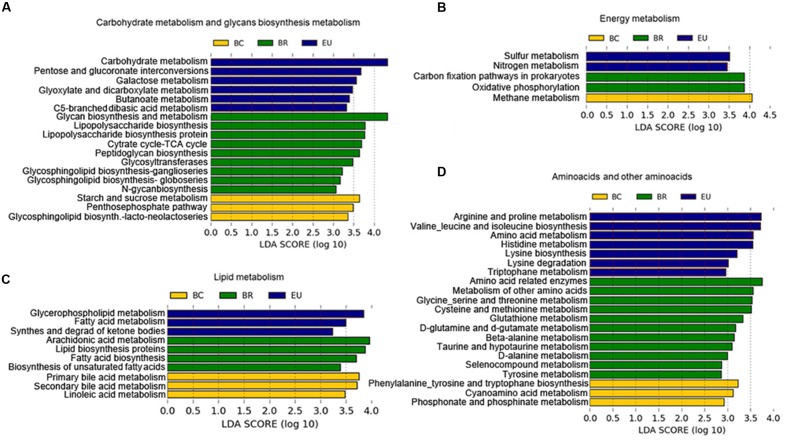
Differences in the most representative bacterial functional classes (KEGG categories level 3). **(A–D)** Functional pathways significantly enriched in African and European populations based on PICRUSt prediction. LEfSe results indicate a sequentially significant ranking among populations (Alpha value = 0.05 for the factorial Kruskal–Wallis test among classes). The threshold for the logarithmic LDA score was 2.0.

The PICRUSt prediction revealed significant differences in the main functional classes [Kyoto Encyclopedia of Genes and Genomes (KEGG) categories at levels 2 and 3], deriving from functional acquisitions associated with different environments and different dietary habits in the four study populations (Supplementary Figure 6). LEfSe analysis (Segata et al., 2011) performed on PICRUSt output showed several KEGG categories differentially present in the African and European populations (Supplementary Figure 6). In particular, considering KEGG function categories at level 3 (**Figure 7**), among the most representative metabolic functions enriched in the BR metagenome, we observed functions involved in complex carbohydrate metabolism, deriving from foods rich in fiber and polysaccharides, such as glycan biosynthesis, glycosyl transferases, and tricarboxylic acid (TCA) cycle (**Figure 7A**). These metabolic functions could explain the microbiota abundance of plant polysaccharides-bacteria degraders and the highest levels of SCFAs observed in fecal samples of BR with respect to other urban populations.

In the BC metagenome, we observed enrichment of KEGG categories involved in starch and sucrose metabolism and methane metabolism (**Figure 7B**), related to the fermentation of polysaccharides (Danielsson et al., 2014). We could hypothesize that the presence of known fermenting bacteria such as *Ruminococcaceae*, *Lachnospiraceae* and *Clostridiaceae*, observed in BC microbiota, can promote functional acquisition related to methane metabolism.

In the EU metagenome, we found enrichment of KEGG functions deriving from a simple sugar-rich diet. The acquisition of functions related to galactose metabolism, involved in conversion of galactose into glucose (**Figure 7A**), could arise from consumption of dairy products. Other functions enriched in the EU gut microbiota were the biosynthesis of carbohydrates from fatty acids and to sulfur and nitrogen metabolism (**Figure 7B**). Several enteric bacteria produce reduced sulfur and nitrogen by dietary amino acids and animal protein. We supposed that bacteria such as *Bilophila*, found abundantly in EU microbiota, a well-known sulfite-reducing bacterium, could be responsible for this functional contribution.

In the BC and EU metagenome, we observed enrichment in functions related to consumption of a diet with high fat and animal protein, such as lipid and amino acid metabolism.

Among the KEGG functional categories, in BC metagenome, we observed enrichment of primary and secondary bile acid biosynthesis (**Figure 7C**). Commensal bacteria deconjugate bile acids, synthesized in the liver from cholesterol-derived precursor molecules, and convert primary into secondary bile acids (Brestoff and Artis, 2013).

In the BR metagenome, PICRUSt analysis showed several enriched amino acid metabolism (**Figure 7D**), suggesting a potential ability of BR microbiota to contribute to host metabolic functions in conditions of poor amino acid food intake. The absence of essential amino acids in food, especially in cereals, which are the basis of the African diet, leads to an inability to synthesize protein and ultimately can lead to malnutrition, especially through a syndrome known as Kwashiorkor, which is common among children in these countries. However, the high intake of cereals and legumes in the BR diet could be a source of amino acids, especially glutamate, alanine, and cysteine. Interestingly, one exceptional source of glycine, alanine, and glutamic acid could be the Baobab, whose leaves are added to main Burkinabè dishes (Supplementary Table 2). Similar findings have been observed in the Hazda, one of the last hunter-gatherer populations, who eat Baobab leaves (Schnorr et al., 2014).

In the BC metagenome, we found enrichment of aromatic amino acid (phenylalanine, tyrosine, and tryptophan; **Figure 7D**), and in the EU, of metabolism of arginine, proline, valine, leucine, isoleucine, histidine and tryptophan, and lysine biosynthesis and degradation, that may originate from the animal protein-rich food, typical of the Western diet (**Figure 7D**). In particular, concerning essential branched-chain amino acid (BCAAs, such as valine, leucine, and isoleucine), our results are in agreement with a recently analyzed metagenome of an Italian population (Rampelli et al., 2015).

The breakdown of basic amino acids by commensal bacteria is a source of SCFAs, as demonstrated by Smith and Macfarlane (1997). In general, alanine, glycine, and cysteine are fermented by bacteria to acetate, propionate, and butyrate. Serine is fermented to acetate and butyrate, while threonine is mainly metabolized to propionate. The main products of methionine metabolism are propionate and butyrate. Interestingly, we found that metabolism of these amino acids was enriched in our BR group, and, together with fiber and polysaccharide fermentation, could contribute to the abundance of SCFAs we observed in their fecal samples (**Figure 3**).

Regarding the more prevalent amino acid metabolism in our EU group, lysine, arginine and deamination of histidine produce butyrate and acetate, while BCAAs are slowly fermented by colonic bacteria. Thus, diet and the functional acquisitions of the bacterial metagenome for metabolism of amino acids or foods rich in fermented fiber and polysaccharides could explain the different levels of SCFAs observed in our studied populations.

## Conclusion

The first years of life are fundamental for acquiring the gut microbial biodiversity, following dietary changes, and are essential for microbiota–host interactions that will later influence the health and disease status in adulthood (Rodriguez et al., 2015).

With respect to our previous work (De Filippo et al., 2010) and other studies investigating dietary habits and gut microbiota composition in traditional populations (Yatsunenko et al., 2012; David et al., 2014; Schnorr et al., 2014; Martinez et al., 2015; Gomez et al., 2016), this preliminary study although performed in a limited groups of children point out the modification of microbiota composition that occurs in African children belonging to the same ethnicity but living in different environments recapitulating processes occurring over a longer time in the co-evolution of diet, gut microbiota and the host.

The gradual enrichment of food variety, with an increase in animal protein (meat, fish, and dairy products), processed and refined foods, the increase in fats and reduction in fiber intake, occurring during the Westernization of dietary habits, drastically changes the microbial profiles and functions, especially bacterial lineages able to ferment complex carbohydrates and produce the well-known anti-inflammatory SCFAs, as observed in urban African children. Despite the limited number of samples from children of each African and Italian population, the dramatic differences among groups presented in this study provide preliminary insights into how improved socio-economic level and exposure to globalized foods could rapidly endanger ancient microbial communities able to ferment dietary fiber, and replaced by other bacteria more suited to metabolism of animal protein, fat and simple sugar. Although the limitations of the approach of functional inference by PICRUSt must be considered, as well as the limited number of individuals analyzed, the concordance of observation on the microbiome and the SCFAs profiles, suggest that the observed differences cannot be explained by chance, but rather from variation in fundamental microbial processes.

These key microbial and metabolic markers that have remained relatively unaltered over 100s of generations in rural Burkina Faso can be considered as reliable indicators of “pristine” microbial patterns, suggesting that dramatic microbial profile losses could occur during the course of urbanization, industrialization, and Westernization.

It is important to remember that children in rural areas of developing countries are at high risk of infectious disease and malnutrition, either because of lack of sanitation and/or the scarcity of food. This has key implications on policies related to mitigation of malnutrition and famine, as well as health assessment and protection of migrants. On the other hand, the gradual disappearance of “old friends” during the transition from rural to urban environments may also be a danger to the health of industrialized and Western populations, in which non-communicable diseases are widespread, as well-reported by hygiene hypothesis (Rook and Brunet, 2005). Our results provides, in a preliminary way, a vital piece to the puzzle of the co-evolution of diet, gut microbiota and host, and highlight the importance of developing strategies to preserve microbial functional acquisitions, especially in childhood, that have been lost during the course of urbanization and the economic development of human populations, and that might have important health implications.

## Additional Information

Data deposition: data were submitted to European Nucleotide Archive (ENA) and available at http://www.ebi.ac.uk/ena/data/view/ERP000133, http://www.ebi.ac.uk/ena/data/view/PRJEB19895.

## Author Contributions

All authors were involved in drafting the article or revising it critically for important intellectual content, and all authors approved the final version to be published. Study conception and design: CDF, PL, DC, and MDP; data analysis: MDP, CDF, MR, DA, GP, and EB; interpretation of data: CDF, MDP, PL, DC, and FM.

## Conflict of Interest Statement

The authors declare that the research was conducted in the absence of any commercial or financial relationships that could be construed as a potential conflict of interest.
