# Supplementary material for: Diet, Environments, and Gut Microbiota. A Preliminary Investigation in Children Living in Rural and Urban Burkina Faso and Italy
Source: Front Microbiol. 2017 Oct 13;8:1979. doi: 10.3389/fmicb.2017.01979 (PMC5645538; doi:10.3389/fmicb.2017.01979)
Supplement: Supplementary file 1 [file Presentation_1.pdf]

Supplementary Material

Diet, environments and gut microbiota. A preliminary investigation in children living in rural and urban Burkina Faso and Italy

Carlotta De Filippo*, Monica Di Paola, Matteo Ramazzotti, Davide Albanese, Giuseppe Pieraccini, Elena Banci, Franco Miglietta, Duccio Cavalieri and Paolo Lionetti*

*Corresponding Author

Correspondence to:

Paolo Lionetti MD, PhD, Department NEUROFARBA, University of Florence, AOU Meyer, Viale Pieraccini 6-24, 50139 - Florence Italy

paolo.lionetti@unifi.it

Carlotta De Filippo, Institute of Biology and Agrarian Biotechnology (IBBA), National Research Council (CNR), Via Moruzzi 1, 56124, Pisa, Italy

carlotta.defilippo@ibba.cnr.it

Supplementary Materials and Methods

Fecal Sample Collection and Bacterial DNA Extraction. Fecal samples were collected by MDs from each individual in the morning, 1-2 hours after the first meal and preserved in RNAlater® (Qiagen) at 4°C for the first 48 hours, and then kept at -80°C until extraction of nucleic acids. The genomic DNA extraction procedure was reported in De Filippo et al 2010 (De Filippo, Cavalieri et al. 2010) and based on a protocol proposed by Zoetendal et al (Zoetendal, Heilig et al. 2006). In brief, after dissolving about 500 mg of each fecal sample in physiological solution and homogenization by vigorous hand shaking, 600 µl of the suspension was centrifuged (10,000 g, for 10 minutes at 4°C) to obtain pellets. The pellets were dissolved in 1 ml ice-cold 1x-PBS and centrifuged at 700 g at 4°C for 1 minute. The supernatants were transferred into a 15 ml tube and were centrifuged at 9000 g at 4°C for 5 minutes. Subsequently, the pellets were suspended in 2.8 ml TE buffer by repeated

pipetting. Then, 180 µl of SDS 10% (w/v) and 18 µl of proteinase K (20 mg/ml) were added. The samples were incubated for 1 hour at 37°C. Afterward, 20 µl RNase (40 µg/ml) were added and incubated at room temperature (RT) for 5 minutes. An equal volume of phenol/chloroform (50:50) was added and the samples were well shaken until the phases were completely mixed. The mixtures were centrifuged at 4500 g for 2 minutes. The upper layers were transferred into a new tube. This step was repeated again so that the interface of the two layers was clean. Then, 1/10 volume of 3 M sodium acetate pH 5.2 and two volumes of 96% ethanol were added and gently mixed. The mixtures were stored overnight at -20°C to precipitate the genomic DNA, then the samples were centrifuged at 4°C at 9000 g for 10 minutes. The genomic DNA was washed twice into 1 ml of 70% ethanol. Finally, dried samples were suspended in 300 µl of nuclease-free water (Ambion). DNA quality was assessed by gel electrophoresis and spectrophotometry measuring OD 260/280. Only samples with good DNA quality were processed.

PCR Amplification of the V5-V6 Region of Bacterial 16S rRNA Genes. For each sample, we amplified 16S rRNA genes using a primer set corresponding to primers 784F and 1061R described by Andersson et al (3). These PCR primers target the V5 and V6 hyper-variable 16S RNA region. The forward primer contained the sequence of the Titanium A adaptor (5'-CCATCTCATCCCTGCGTGTCTCCGACTCAG-3') and a barcode sequence. For each sample, a PCR mix of 100 µl was prepared containing 1x PCR buffer, 5U of FastStart High Fidelity polymerase blend and dNTPs from the FastStart High Fidelity PCR system (Roche), 200 nM of primers (Eurogentec) and 100 ng of gDNA. Thermal cycling consisted of initial denaturation at 95°C for 5 minutes followed by 30 cycles of denaturation at 95°C for 40 seconds, annealing at 55°C for 40 seconds, and extension at 72°C for 1 minute, with a final extension of 7 minutes at 72°C. Amplicons were visualized on 1.0% agarose gels using SYBR Safe DNA gel stain in 0.5x TBE (Invitrogen) and were cleaned using the HighPure Cleanup kit (Roche) according to the manufacturer’s instructions.

Amplicon Quantitation, Pooling and Pyrosequencing. Amplicon DNA concentrations were determined using the Quant-iT PicoGreen dsDNA reagent and kit (Invitrogen) following the manufacturer's instructions. Assays were carried out using 10 µl of cleaned PCR product in a total reaction volume of 200 µl in black, 96-well microtiter plates. Fluorescence was measured on Perkin Elmer Victor Plate reader using the 485/530 nm excitation/emission filter pair with measurement time 0.1 second. Following quantitation, cleaned amplicons were combined in equimolar ratios into a single tube. The final pool of DNA was precipitated on ice for 45 minutes following the addition of 5 M NaCl (0.2 M final concentration) and two volumes of ice-cold 100% ethanol. The precipitated DNA was centrifuged at 7,800 g for 40 minutes at 4°C, and the resulting pellet was washed with an equal volume of ice-cold 70% ethanol and centrifuged again at 7,800 g for 20 minutes at 4°C. The supernatant was removed and the pellet was air dried for 10 minutes at room temperature and then resuspended in 100 µl of nuclease-free water (Ambion). The final concentration of the pooled DNA was determined using a NanoDrop spectrophotometer (Thermo Fisher). Pyrosequencing was carried out using primer A on a 454 Life Sciences Genome Sequencer FLX instrument (Roche) following Titanium chemistry.

Data Analysis. Data analysis integrated together the previous data obtained for BR and EU populations (De Filippo, Cavalieri et al. 2010) with data from BT and EU samples. We obtained 256`308 filtered reads for BR samples (Number of reads for each sample, mean 23`300.73; Read length, mean 235.73) and 303`972 filtered reads for EU Samples (Number of reads for each sample, mean 23`382.46; Read length, mean 235.95). For BT samples 194`202 filtered reads (Number of reads for each BT sample, mean 24`275.25; Read length, mean 244.64), and 89`888 filtered reads for BC (Number of reads for each BC sample, mean 17`977.6; Read length, mean 291) were obtained. Raw 454 files were demultiplexed using Roche's .sff file software.

Reads of all data sets were pre-processed using the MICCA pipeline (version 1.5, http://compmetagen.github.io/micca/) (Albanese, Fontana et al. 2015). Forward and reverse primer trimming and quality filtering were performed using micca-preproc truncating reads shorter than 280nt (quality threshold=18). Denovo sequence clustering, chimera filtering and taxonomy assignment were performed by micca-otu-denovo (parameters -s 0.97 -c). Operational Taxonomic Units (OTUs) were assigned by clustering the sequences with a threshold of 97% pair-wise identity, and their representative sequences were classified using the RDP software version 2.7 (Wang, Garrity et al. 2007). Template-guided multiple sequence alignment was performed using PyNAST57 (version 0.1) (Caporaso, Bittinger et al. 2010) against the multiple alignment of the Greengenes 16S rRNA gene database (DeSantis, Hugenholtz et al. 2006) filtered at 97% similarity. Finally, a phylogenetic tree was inferred using FastTree (Price, Dehal et al. 2010) and micca-phylogeny (parameters: -a template-template-min-perc 50). Sampling heterogeneity was reduced by rarefaction, obtaining 12`964 sequences per sample.

Bacterial species were assigned, based on Basic Local Alignment Search Tool nucleotide (BLASTn) software in the National Center for Biotechnology Information (NCBI) database, considering the highest percentage of identity (Query cover 100%-99% and Identity 99% or 95%). Expectation value (E-value) was used to select significant BLAST hits, keeping only outcomes with the lowest E-value, given a minimal E-value of 10^{-3} (generally, significant match when the E-value is close to zero).Chao1 index and Shannon entropy (indicators of alpha diversity) and UniFrac (Lozupone, Lladser et al. 2011) and Bray-Curtis dissimilarities (indicators of beta diversity) were calculated using the phyloseq package (McMurdie and Holmes 2014) of the R software suite. Exploratory analysis was performed by Principal coordinates analysis (PCoA) using the phyloseq package of the R software suite. Multiple-rarefaction PCoA plots ("jackknifed" PCoA plots) (Lozupone, Lladser et al. 2011) were computed to assess the robustness of the beta-diversity analyses.

The significance of between-groups differentiation on the UniFrac distances and Bray-Curtis dissimilarity was assessed by PERMANOVA using the adonis() function of the R package vegan

with 999 permutations.

To compare the relative abundances of OTUs among the four groups, the two-sided, unpaired Wilcoxon test was computed, removing taxa not having a relative abundance of at least 0.1%, in at least 20% of the samples, and using the function mt() in the phyloseq library and the p-values were adjusted for multiple comparison controlling the family-wise Type I error rate (minP procedure).

Based on sequence abundances in each population, heatmap plots of percentage abundances, at different taxa, were obtained by using STAMP (Parks, Tyson et al. 2014), and supported by dendogram, obtained with Average Neighbour and Unweighted Pair Group Method with Arithmetic Mean (UPGMA), useful to cluster fecal samples of the children populations based on taxa abundances.

Based on the relative abundances, the metagenomic biomarker discovery and related statistical significance were assessed using the linear discriminant analysis (LDA) effect size (LEfSe) method (Segata, Izard et al. 2011). LEfSe uses the Kruskal–Wallis rank-sum test to identify features with significantly different abundances between assigned taxa compared to the groups, and LDA to estimate the size effect of each feature. An alpha significance level of 0.05, either for the factorial Kruskal-Wallis test among classes or for the pairwise Wilcoxon test between subclasses, was used. A size-effect threshold of 2.0 on the logarithmic LDA score was used for discriminative microbial biomarkers.

To infer the functional contribution of microbial communities on 16S rDNA sequencing data set, we applied PICRUSt (Phylogenetic Investigation of Communities by Reconstruction of Unobserved States) (Langille, Zaneveld et al. 2013), that implements an extended ancestral-state reconstruction algorithm to predict which gene families are present, and then combines gene families to estimate the significant differences in the main functional classes (KEGG categories) of the composite metagenome. From a OTUs table with associated Greengenes identifiers, we obtained the final output from metagenome prediction as an annotated table of predicted gene family counts for each sample, where the encoded functions of each gene family are orthologous groups or other identifiers such as

KEGG orthologs (KOs). The functional pathways discovery and related statistical significance were assessed by LEfSe.

In general, PICRUSt maps the subset of 16S sequences to their nearest sequenced reference genome. To evaluate accuracy of PICRUSt, we used the Nearest Sequenced Taxon Index (NSTI), developed to quantify the availability of nearby genome representatives for each microbiome sample (Supplementary Table 6).

Determination of Short-Chain Fatty Acids (SCFAs) in Fecal Samples. For determination of SCFASs we used an aliquot of frozen fecal samples (about 250 mg). Briefly, fecal samples were homogenized after addition of 1 ml of 10% perchloric acid and centrifuged at 15,000 g for 5 minutes at 4°C. Concentrations of SCFASs were determined in a 1:25 dilution of 500 µl supernatant. We used 5 µl of a mixture of deuterated acids containing 50 ng D3-propionic, 50 ng D7-butyric and 500 ng D4-acetic acids as internal standard. A calibration curve was prepared, adding the mixture of internal standards (5 µl) to scalar amounts of the acids. SPME-GC-MS determinations were performed using a Varian Saturn 2000 GC-MS instrument with 8200 CX SPME autosampler. The SPME fiber was a Carboxen/Divinylbenzene 75 µm. The capillary column was an Agilent HP-Innowax 30 m × 0.25 mm, 0.5 µm film thickness. The injector and transfer line temperatures were 290°C and 260°C, respectively; the ion trap temperature was 180°C. Absorption of analytes was performed in the headspace of the sample solution for 3 min at 70°C; the analytes were desorbed in the GC injector port at 290°C for 20 min. The GC oven temperature program was as follows: initial temperature 45°C for 0.15 min, then to 123°C at 2°C/min, to 159°C at 6°C/min and to 200°C at 20°C/min. The retention times for individual short-chain fatty acids were determined by injecting each standard into the column. The Varian MS workstation software (version 6.6) was used for data acquisition and processing. The SCFAs concentration in fecal sample was expressed in µmol/g of feces. To determine statistical significance of differences observed among the four populations we used unpaired

Student's t test (one tailed).

Supplementary Results

Microbiota characterization of African children populations compared to Europeans

Among the minor components of gut microbiota (relative abundance <0.05), we observed that *Coriobacteriaceae* was significantly enriched in BT compared to EU, and *Erysipelotrichaceae* in BC compared with BR and EU (Supplementary Figure 5; Wilcoxon rank-sum test; Supplementary Table 5B). *Leuconostocaceae* was significantly more abundant in BR compared with EU (Supplementary Figure 5; Wilcoxon rank-sum test).

Bacterial species assignment

By BLAST alignment of 16S sequences, we found that the majority of sequences belonging to *Bacteroides* genus and consistently found in BC and EU metagenome, was attributable to *B. uniformis*, and in minor abundance to *B. acidifaciens*, *B. caccae*, *B. coprophilus*, *B. ovatus*, and *B. plebeius*. In BR and BT populations, although *Bacteroides* was poorly represented, we found *Bacteroides* sequences attributable with 95% of identity to *B. vulgatus*. Interestingly, a study on gnotobiotic interleukin-2-deficient mice showed that *B. vulgatus* has a protective role against *E. coli* induced-colitis (Waidmann, Bechtold et al. 2003), suggesting that the probable unique *Bacteroides* species found in BR microbiota could have a possible protective role against potential pathogenic bacteria.

Regarding *Bifidobacterium* genus, we observed that BC and EU populations were mainly enriched in *B. longum,* and in minor part in *B. adolescentis*, *B. bifidum* and *B. breve*.

Functional metabolic profiles of gut microbiota by PICRUSt

In order to evaluate how the observed taxonomic differences between the gut microbiota of African and European children affect their metabolic potential, we applied PICRUSt (Phylogenetic Investigation of Communities by Reconstruction of Unobserved States), a computational approach useful to infer the functional contribution of microbial communities on 16S rDNA sequencing data set. NSTI values (Supplementary Table 6) calculated by 16S data set of the four children populations (a measure of the accuracy of PICRUSt prediction based on phylogenetic distances nearest sequenced reference genome for each microorganism) showed comparable values between BR and BT populations (mean NSTI=0.07 ±0.009 s.d. and NSTI=0.07 ±0.03 s.d. in BR and BT) and between BC and EU populations (mean NSTI=0.10 ±0.01 s.d. and NSTI=0.11 ±0.01 s.d. respectively).

We decided to use this approach based on 16S rRNA inference, instead of whole genome sequencing, although we are aware that probably several microbial functions related to metagenome of isolated and traditional populations could be still unknown. Despite this potential limitation, PICRUSt prediction performed on samples from Burkina Faso and European children revealed significant differences in the main functional classes (KEGG categories at level 2), deriving from functional acquisitions associated to the different environments and to different dietary habits in the four populations (Supplementary Figure 6A). LEfSe analysis performed on PICRUSt output showed several KEGG categories differentially enriched in the African and European populations (Supplementary Figure 6A).

Carbohydrate Metabolism

In the BR metagenome, we found enrichment of carbon fixation pathways and oxidative phosphorylation, metabolic functions that are both related to carbohydrate metabolism and involved in releasing energy, associated with the TCA cycle pathway (Figure 7A-B).

In the BC metagenome, we observed enrichment of starch and sucrose metabolism, as well as pentose phosphate metabolism, a metabolic pathway parallel to glycolysis that generates NADPH and ribose 5-phosphate, a precursor for the synthesis of nucleotides, and erythrose 4-phosphate (E4P), used in

the synthesis of aromatic amino acids. Interestingly, the BC metagenome was also enriched in methane metabolism (Figure 7B), related to the fermentation of polysaccharides (Danielsson, Werner-Omazic et al. 2014).

In the EU metagenome, over the general pathway related to carbohydrate metabolism, pentose, glucuronate interconversion pathways and C5-branched dibasic acid metabolism, deriving from a simple sugar-rich diet, we found enrichment of galactose metabolism, involved in conversion of galactose into glucose (Figure 7A). The acquisition of these functions in the EU metagenome could arise from consumption of dairy products. Other functions enriched in the EU gut microbiota were glyoxylate and dicarboxylate metabolism, involved in the biosynthesis of carbohydrates from fatty acids. Sulfur and nitrogen metabolism were also enriched in the EU metagenome (Figure 7B). Regarding nitrogen metabolism, several enteric bacteria produce reduced sulfur and nitrogen by dietary amino acids and animal protein.

Lipid Metabolism

Studies on animal models have shown that commensal bacteria can regulate and maintain lipid homeostasis (Brestoff and Artis 2013). Our results reveal differential functional lipid metabolism characterized the metagenomes of the four studied populations (Figure 7C). In BR children, we found enrichment of arachidonic acid, fatty acid biosynthesis and lipid biosynthesis proteins (Figure 7C).

In BC children, we observed enrichment of linoleic metabolism, an essential polyunsaturated fatty acid involved in the biosynthesis of arachidonic acid, a key inflammatory intermediate. We also found enrichment of primary and secondary bile acid biosynthesis (Figure 7C). The synthesis of bile acids is one of the predominant mechanisms for the excretion of excess cholesterol, and is involved in intestinal absorption of fat-soluble vitamins.

In EU children we found an enrichment of glycerophospholipid and fatty acid metabolism and synthesis and degradation of ketone bodies (Figure 7C), clearly derived from a lipid-rich diet. The

synthesis and degradation of ketone bodies could be derived from glucose production from non-carbohydrate sources, and could explain the observed enrichment in butanoate metabolism.

Amino acid Metabolism

In the BR metagenome, PICRUSt analysis showed several enriched amino acid metabolism (Figure 7D). Commensal bacteria can provide amino acids to the host from both dietary and endogenous proteins. Peptides and amino acids are used as carbon, nitrogen and energy sources by both saccharolytic and non-saccharolytic bacteria. Some saccharolytic species, such as *Prevotella*, the predominant genus in BR children, are able to derive energy from the carbon skeletons of peptides and amino acids.

Glutamate is the principal source of nitrogen for the host. Bacteria can make the carbon skeletons of all amino acids and transaminate those carbon skeletons with nitrogen from glutamine or glutamate to complete the amino acid structures (Berg, Tymoczko et al., 2002). Interestingly, the observed enrichment in beta and D-alanine metabolism represents a way to produce glucose, especially in fasting conditions. Alanine can then be converted to pyruvate in the liver by the glucose-alanine cycle, as a source of carbon for gluconeogenesis, in order to form glucose that can be used as energy for the muscle.

Another metabolic function found in the BR population is seleno-compound metabolism, deriving from consumption of cereal grains, legumes and soybeans (Whanger 2002) (Figure 7D). Seleno compounds are organometallic molecules comprised of selenium, an essential element involved in reproduction, thyroid hormone metabolism, DNA synthesis, and protection from oxidative damage and infection (Sunde and Thompson 2009).

Surprisingly, metabolism of taurine and hypotaurine was enriched in the BR metagenome (Figure 7D). Generally, taurine is a major constituent of bile acid, important in emulsifying fat. However, taurine is also derived from cysteine, thus enrichment of cysteine metabolism found in BR children could explain this functional acquisition.

In the BC metagenome, we found enrichment of aromatic amino acid (phenylalanine, tyrosine and tryptophan; Figure 7D). In BC children, we also found enrichment of phosphonate and phosphinate metabolism (Figure 7D), related to carbon-phosphorous bonds (C-P compounds) biosynthesized by Actinobacteria that were abundant in BC microbiota.

In the EU metagenome, we observed enrichment of metabolism of amino acids such as arginine, proline, valine, leucine, isoleucine, histidine and tryptophan, and lysine biosynthesis and degradation (Figure 7D). These amino acids can originate from the animal protein-rich food, typical of the Western diet. Concerning essential branched-chain amino acid (BCAAs, such as valine, leucine, and isoleucine) our results are in agreement with a recently analyzed metagenome of an Italian population (Rampelli, Schnorr et al. 2015). It is noteworthy that metabolic function related to BCAAs and aromatic amino acids are enriched in obese compared to lean individuals (Newgard, An et al. 2009), and increased BCAA levels are associated with the risk of developing type 2 diabetes (Wang, Larson et al. 2011).

Cofactors and Vitamin metabolism

Commensal colonic bacteria are a significant source of a range of vitamins to the host (Hill 1997). Unlike dietary vitamins, the uptake of vitamins derived by microbial metabolism predominantly occurs in the colon (Said and Mohammed 2006). Among this, the B vitamins, including thiamin, riboflavin, niacin, folate, vitamin B6, vitamin B12, biotin and pantothenic acid, cooperate to perform many different processes, such as the release of energy deriving from carbohydrates, proteins and fats, or regulation of immune cells (Brestoff and Artis 2013). The infant microbiota appears to be specialized for the acquisition of nutrients, especially the vitamin B (Yatsunenko, Rey et al. 2012). In BR metagenome, we found several functional acquisitions related to cofactors and coenzyme involved in oxidative reactions, energy, and carbohydrate and amino acids metabolism, and for DNA and RNA building (Supplementary Figure 6B), such as folate biosynthesis, riboflavin, vitamin B6, retinol metabolism, nicotinate and nicotinamide metabolism. In BC metagenome, lipoic acid and

biotin metabolism were enriched (Supplementary Figure 6B). The former is an important cofactor for mitochondrial enzyme complexes in antioxidant reactions. The latter is a coenzyme for carboxylase enzymes, involved in the synthesis of fatty acids, isoleucine, and valine, and in gluconeogenesis.
In EU, we found enrichment in functions related to porphyrin and chlorophyll metabolism (porphyrins are essential cofactors of many proteins including cytochrome, haemoglobin and myoglobin), panthotenate and CoA metabolism, and thiamine, a coenzyme involved in the catabolism of sugars and aminoacids (Supplementary Figure 6B).

Secondary metabolisms

Human-associated bacteria produce also a wide range of natural compounds, such as terpenoids and polyketides (Donia and Fischbach 2015), that include well-characterized mediators of microbe-host and microbe-microbe interactions. In the BR microbiome, we observed a great enrichment of functions related to metabolism of terpenoids, probably due to a high consumption of plant-derived foods, and polyketides (Supplementary Figure 6D). Among the metabolic functions related to biosynthesis of polyketides, many of the commonly used antibiotics, such as tetracycline and macrolides, are produced by polyketide synthases. In the four groups of children, we observed differentially and progressively increased antibiotic biosynthesis functions, passing from rural to urban populations. In particular, we found acquired streptomycin biosynthesis function in the BR metagenome; novobiocin biosynthesis in the BT group; butirosin, neomycin and ansamycin biosynthesis in the BC; and beta-lactamase resistance, penicillin and cephalosporin biosynthesis, and tetracycline biosynthesis in the EU children (Supplementary Figure 6C-D). In a rural environment, antibiotic production by bacteria is a competition mechanism that could provide selective benefit for the producing microorganism. Most antibiotics are derived from biomolecules and secondary metabolites produced by soil-dwelling microorganisms (Davies and Davies 2010); therefore microbiota of rural populations may be acquiring this potential for the unique scope of survival and competition among bacteria. The observed beta-lactamase resistance and cephalosporin biosynthesis

acquisitions in the EU metagenome confirm that the use of antibiotics in agriculture, in livestock and medical practice are inducing functional acquisitions related to antibiotic resistance, an emerging and dramatic problem in industrialized and globalized populations.

Regarding the xenobiotics metabolism, in the BR metagenome, we found enrichment of aromatic organic compounds, such as toluene, naphthalene and ethylbenzene degradation (Supplementary Figure 6E). This pathway referred to several compounds containing the benzene ring, including phenolic molecules. As observed in our recent study on functional acquisitions related to xenobiotic degradation by microbiota of red colobus monkeys eating several plant species in the forest of Tanzania (Barelli, Albanese et al. 2015), we may hypothesize that the widespread presence of plants and vegetables rich in tannins and phenolic compounds in rural villages of Burkina Faso, require metabolic acquisition of functions for digestion of xenobiotics by commensal bacteria, rather than the acquired functions related to environmental contaminant degradation.

In urban BC and EU metagenomes, we found enrichment of chloralkene, bisphenol, dioxin and xylene degradation, and benzoate and 1,1,1-trichloro-2,2-bis(4-chlorophenyl)-ethane-(DDT) degradation, respectively (Supplementary Figure 6E). The degradation of these xenobiotics by the microbiota of urban populations can be considered a functional response of bacteria to exposure to toxic compounds derived from industrial and urban environmental pollution (especially chloroalkene and dioxin). Among benzoate compounds, sodium benzoate, a food preservative known as E211, is widely used in acidic foods, carbonated drinks, jams and fruit juices. Furthermore, several chlorinated organic insecticides are persistent contaminants in the urban environment and potentially in cultivated foods.

References

Albanese, D., P. Fontana, C. De Filippo, D. Cavalieri and C. Donati (2015). MICCA: a complete and accurate software for taxonomic profiling of metagenomic data. *Sci Rep* **5**: 9743.

Barelli, C., D. Albanese, C. Donati, M. Pindo, C. Dallago, F. Rovero, D. Cavalieri, K. M. Tuohy, H. C. Hauffe and C. De Filippo (2015). Habitat fragmentation is associated to gut microbiota diversity of an endangered primate: implications for conservation. Sci Rep 5: 14862.

Brestoff, J. R. and D. Artis (2013). Commensal bacteria at the interface of host metabolism and the immune system. *Nat Immunol* **14**(7): 676-684.

Berg, J.M., Tymoczko, J.L. and L. Stryer (2002). Biochemistry: International Edition Hardcover, 5th edition.

Caporaso, J. G., K. Bittinger, F. D. Bushman, T. Z. DeSantis, G. L. Andersen and R. Knight (2010). PyNAST: a flexible tool for aligning sequences to a template alignment. *Bioinformatics* **26**(2): 266-267.Danielsson, R., A. Werner-Omazic, M. Ramin, A. Schnurer, M. Griinari, J. Dicksved and J. Bertilsson (2014). Effects on enteric methane production and bacterial and archaeal communities by the addition of cashew nut shell extract or glycerol-an in vitro evaluation. J Dairy Sci 97(9): 5729-5741.

Davies, J. and D. Davies (2010). Origins and evolution of antibiotic resistance. Microbiol Mol Biol Rev 74(3): 417-433.

De Filippo, C., D. Cavalieri, M. Di Paola, M. Ramazzotti, J. B. Poullet, S. Massart, S. Collini, G. Pieraccini and P. Lionetti (2010). Impact of diet in shaping gut microbiota revealed by a comparative study in children from Europe and rural Africa. *Proc Natl Acad Sci U S A* **107**(33): 14691-14696.

DeSantis, T. Z., P. Hugenholtz, N. Larsen, M. Rojas, E. L. Brodie, K. Keller, T. Huber, D. Dalevi, P. Hu and G. L. Andersen (2006). Greengenes, a chimera-checked 16S rRNA gene database and workbench compatible with ARB. *Appl Environ Microbiol* **72**(7): 5069-5072.

Donia, M. S. and M. A. Fischbach (2015). HUMAN MICROBIOTA. Small molecules from the human microbiota. Science 349(6246): 1254766.

Hill, M. J. (1997). Intestinal flora and endogenous vitamin synthesis. *Eur J Cancer Prev* **6 Suppl 1**: S43-45.

Langille, M. G., J. Zaneveld, J. G. Caporaso, D. McDonald, D. Knights, J. A. Reyes, J. C. Clemente, D. E. Burkepile, R. L. Vega Thurber, R. Knight, R. G. Beiko and C. Huttenhower (2013). Predictive functional profiling of microbial communities using 16S rRNA marker gene sequences. *Nat Biotechnol* **31**(9): 814-821.

Lozupone, C., M. E. Lladser, D. Knights, J. Stombaugh and R. Knight (2011). UniFrac: an effective distance metric for microbial community comparison. *Isme Journal* **5**(2): 169-172.

McMurdie, P. J. and S. Holmes (2014). Waste not, want not: why rarefying microbiome data is inadmissible. *PLoS Comput Biol* **10**(4): e1003531.

Newgard, C. B., J. An, J. R. Bain, M. J. Muehlbauer, R. D. Stevens, L. F. Lien, A. M. Haqq, S. H. Shah, M. Arlotto, C. A. Slentz, J. Rochon, D. Gallup, O. Ilkayeva, B. R. Wenner, W. S. Yancy, Jr., H. Eisenson, G. Musante, R. S. Surwit, D. S. Millington, M. D. Butler and L. P. Svetkey (2009). A branched-chain amino acid-related metabolic signature that differentiates obese and lean humans and contributes to insulin resistance. Cell Metab 9(4): 311-326.

Parks, D. H., G. W. Tyson, P. Hugenholtz and R. G. Beiko (2014). STAMP: statistical analysis of taxonomic and functional profiles. *Bioinformatics* **30**(21): 3123-3124.

Price, M. N., P. S. Dehal and A. P. Arkin (2010). FastTree 2--approximately maximum-likelihood trees for large alignments. *PLoS One* **5**(3): e9490.

Rampelli, S., S. L. Schnorr, C. Consolandi, S. Turroni, M. Severgnini, C. Peano, P. Brigidi, A. N. Crittenden, A. G. Henry and M. Candela (2015). Metagenome Sequencing of the Hadza Hunter-Gatherer Gut Microbiota. Curr Biol 25(13): 1682-1693.

Said, H. M. and Z. M. Mohammed (2006). Intestinal absorption of water-soluble vitamins: an update. *Curr Opin Gastroenterol* **22**(2): 140-146.

Segata, N., J. Izard, L. Waldron, D. Gevers, L. Miropolsky, W. S. Garrett and C. Huttenhower (2011). Metagenomic biomarker discovery and explanation. *Genome Biol* **12**(6): R60.

Sunde, R. A. and K. M. Thompson (2009). Dietary selenium requirements based on tissue selenium concentration and glutathione peroxidase activities in old female rats. J Trace Elem Med Biol 23(2): 132-137.

Waidmann, M., O. Bechtold, J. S. Frick, H. A. Lehr, S. Schubert, U. Dobrindt, J. Loeffler, E. Bohn and I. B. Autenrieth (2003). Bacteroides vulgatus protects against Escherichia coli-induced colitis in gnotobiotic interleukin-2-deficient mice. *Gastroenterology* **125**(1): 162-177.

Wang, Q., G. M. Garrity, J. M. Tiedje and J. R. Cole (2007). Naive Bayesian classifier for rapid assignment of rRNA sequences into the new bacterial taxonomy. *Appl Environ Microbiol* **73**(16): 5261-5267.

Wang, T. J., M. G. Larson, R. S. Vasan, S. Cheng, E. P. Rhee, E. McCabe, G. D. Lewis, C. S. Fox, P. F. Jacques, C. Fernandez, C. J. O'Donnell, S. A. Carr, V. K. Mootha, J. C. Florez, A. Souza, O. Melander, C. B. Clish and R. E. Gerszten (2011). Metabolite profiles and the risk of developing diabetes. Nat Med 17(4): 448-453.

Whanger, P. D. (2002). Selenocompounds in plants and animals and their biological significance. J Am Coll Nutr 21(3): 223-232.

Yatsunenko, T., F. E. Rey, M. J. Manary, I. Trehan, M. G. Dominguez-Bello, M. Contreras, M. Magris, G. Hidalgo, R. N. Baldassano, A. P. Anokhin, A. C. Heath, B. Warner, J. Reeder, J. Kuczynski, J. G. Caporaso, C. A. Lozupone, C. Lauber, J. C. Clemente, D. Knights, R. Knight and J. I. Gordon (2012). Human gut microbiome viewed across age and geography. *Nature* **486**(7402): 222-227.

Zoetendal, E. G., H. G. Heilig, E. S. Klaassens, C. C. Booijink, M. Kleerebezem, H. Smidt and W. M. de Vos (2006). Isolation of DNA from bacterial samples of the human gastrointestinal tract. *Nat Protoc* **1**(2): 870-873.

Supplementary Table

Supplementary Table 1. Characteristics of the four children populations

Sample ID	Group	Nation	Environment	Ethnicity	Mode of birth	Sex	Age
2BR	BR	Burkina Faso	Rural village	Mossi	Natural	M	5
6BR	BR	Burkina Faso	Rural village	Mossi	Natural	F	6
7BR	BR	Burkina Faso	Rural village	Mossi	Natural	M	6
8BR	BR	Burkina Faso	Rural village	Mossi	Natural	M	6
9BR	BR	Burkina Faso	Rural village	Mossi	Natural	M	6
10BR	BR	Burkina Faso	Rural village	Mossi	Natural	F	6
11BR	BR	Burkina Faso	Rural village	Mossi	Natural	M	5
12BR	BR	Burkina Faso	Rural village	Mossi	Natural	M	6
13BR	BR	Burkina Faso	Rural village	Mossi	Natural	M	6
15BR	BR	Burkina Faso	Rural village	Mossi	Natural	M	6
17BR	BR	Burkina Faso	Rural village	Mossi	Natural	F	5
						ratio (F:M) 3:8	age (average ±SD) 5.7 ±0.46
1BT	BT	Burkina Faso	Small Town	Mossi	Natural	M	2
2BT	BT	Burkina Faso	Small Town	Mossi	Natural	F	3
3BT	BT	Burkina Faso	Small Town	Mossi	Natural	F	8
4BT	BT	Burkina Faso	Small Town	Mossi	Natural	M	4
5BT	BT	Burkina Faso	Small Town	Mossi	Natural	M	5
6BT	BT	Burkina Faso	Small Town	Mossi	Natural	M	4
7BT	BT	Burkina Faso	Small Town	Mossi	Natural	M	7
8BT	BT	Burkina Faso	Small Town	Mossi	Natural	F	2
						ratio (F:M) 3:5	age (average ±SD) 4.4±2.19
1BC	BC	Burkina Faso	Capital city	Mossi	Natural	M	4
2BC	BC	Burkina Faso	Capital city	Mossi	Natural	F	3
3BC	BC	Burkina Faso	Capital city	Mossi	Natural	F	2
4BC	BC	Burkina Faso	Capital city	Mossi	Natural	F	3
5BC	BC	Burkina Faso	Capital city	Mossi	Natural	F	2
						ratio (F:M) 4:1	age (average ±SD) 2.8±0.83

1EU	EU	Italy	European city (Florence)	Caucasian	Cesarean childbirth	M	2
5EU	EU	Italy	European city (Florence)	Caucasian	Natural	M	5
6EU	EU	Italy	European city (Florence)	Caucasian	Natural	M	6
8EU	EU	Italy	European city (Florence)	Caucasian	Natural	M	5
10EU	EU	Italy	European city (Florence)	Caucasian	Natural	M	5
11EU	EU	Italy	European city (Florence)	Caucasian	Natural	M	5
12EU	EU	Italy	European city (Florence)	Caucasian	Natural	M	6
13EU	EU	Italy	European city (Florence)	Caucasian	Natural	M	5
17EU	EU	Italy	European city (Florence)	Caucasian	Natural	M	5
18EU	EU	Italy	European city (Florence)	Caucasian	Natural	F	3
19EU	EU	Italy	European city (Florence)	Caucasian	Natural	F	4
20EU	EU	Italy	European city (Florence)	Caucasian	Natural	F	5
21EU	EU	Italy	European city (Florence)	Caucasian	Cesarean childbirth	F	3
						ratio (F:M) 4:9	age (average ±SD) 4.5±1.19
Total groups						ratio (F:M)14:23	age (average ±SD) 4.6±1.55

Supplementary Table 2. Total daily food intake in terms of protein, fat, carbohydrate and fiber in relation to the average of maximum quantity ingested per day relative to Burkina Faso and European children.

(A)

BR	Composition of Edible Portion							
**Dish component**	**Daily Q (grams)***	**Percentage on total daily Q**	**Food Energy (Kcal)**	**Moisture (%)**	**Protein (g)**	**Fat (g)**	**Carbohydrate, total (incl. fiber) (g)**	**Fiber (g)**
**Cereals and starchy component (Millet, Sorghum)**	170	38%	495.4	46.38	10.1	4.06	105.53	1.09
**Legumes (Niebè, Black-Eyed Peas)**	70	16%	267.4	6.02	25.795	8.47	26.285	6.16
**Vegetables (Nerè)**	60	13%	55.2	47.1	3.36	3.84	4.26	0.612
**Fruit (mango, papaya)**	130	29%	48.75	115.7	0.91	0.195	12.48	6.37
**Milk and Milk derivatives**								
**Meats, meat derivatives, fish**								
**Egg**								
**Oil and fats (karitè)**	15	3%	129.3	0.21	0	14.67	0.09	0
**Peanuts**								
**Sugar and honey**								
**Total daily food intake**	**445**		**996.1**		**40.2**	**31.2**	**148.6**	**14.2**

* Average of max Quantity ingested per child per day

(B)

BT	Composition of Edible Portion							
**Dish component**	**Daily Q (grams)***	**Percentage on total daily Q**	**Food Energy (Kcal)**	**Moisture (%)**	**Protein (g)**	**Fat (g)**	**Carbohydrate, total (incl. fiber) (g)**	**Fiber (g)**
**Cereals and starchy component (Millet, Sorghum, Rice, Corn)**	195	42.4%	470	78	10.4	4.0	100.4	1.6
**Legumes (Niebè, Black-Eyed Peas)**	70	15.2%	222.6	4.9	21.2	7.0	21.8	4.9
**Vegetables (Nerè and Baobab leaves)**	50	10.9%	136	21.6	8.8	9	8	1
**Fruit (mango, papaya)**	100	21.7%	37.5	89.2	0.6	0.1	9.6	4.8
**Milk and Milk derivatives**								
**Meats, meat derivatives, fish (mutton**; lake fish**)**	20	4.3%	32.9	11.3	3.71	0.58	0.02	0
**Egg**								
**Oil**	15	3.3%	135	0	0	15	0	0
**Peanuts**	10	2.2%	60.5	0.5	1.7	5.5	2.2	0.2
**Sugar and honey**								

Total daily food intake	460.0		1094.5		46.5	41.2	142	12.5

* Average of max Quantity ingested per child per day

**once a week

(C)

BC	Composition of Edible Portion							
**Dish component**	**Daily Q (grams)***	**percentage on total daily Q**	**Food Energy (Kcal/die)**	**Moisture (%)**	**Protein (g)**	**Fat (g)**	**Carbohydrate, total (incl. fiber) (g)**	**Fiber (g)**
**Cereals and starchy component (bread, millet, sorghum, corn)**	160	17.1%	384	40	8.5	3.2	82.4	1.4
**Legumes**	30	3.2%	95.4	26.25	9.2	15	9.4	2.1
**Vegetables (Nerè, tomatoes, carrots, zucchini, aubergine)**	120	12.8%	10.8	49.39	1.49	0.17	2.52	1.1
**Fruit (mango, bananas, papaya)**	100	10.7%	37.5	89.2	0.6	0.1	9.6	4.8
**Fruit juice**	110	11.8%	53.9	40.66	0.55	0.09	13.9	0
**Milk and Milk derivatives (cow's milk, yoghurt, cheese)**	250	26.7%	246.7	218.5	12	16.65	13.2	0
**Meats, meat derivatives, fish**	60	6.4%	98.8	34.14	11.13	1.75	0.06	0
**Egg**	30	3.2%	38.4	77.1	3.72	2.61	0	0
**Oil**	20	2.1%	180	0	0	20	0	0
**Peanuts**	15	1.6%	90.7	7.5	2.55	8.25	3.3	0.3
**Sugar and honey**	10	1.1%	38.7	0	0	0	10	0
**Snacks**	30	3.2%	179.55	18.8	2.2	8.7	34.9	0
**Total daily food intake**	**935**		**1454.27**		**51.92**	**76.5**	**179.26**	**9.7**

* Average of max Quantity ingested per child per day

(D)

EU	Composition of Edible Portion							
**Dish component**	**Daily Q (grams)***	**percentage on total daily Q**	**Food Energy (Kcal/die)**	**Moisture (%)**	**Protein (g)**	**Fat (g)**	**Carbohydrate, total (incl. fiber) (g)**	**Fiber (g)**
**Cereals and starchy component**	160	17.3%	375	68.02	9.7	4.89	152.38	3.46
**Legumes (Beans, String beans, Peas)**	20	2.2%	10.9	15.4	1.2	0.1	3.7	0.9
**Vegetables (carrot, potatoes, fennel, tomato, zucchini)**	100	10.8%	50.9	82.3	2.3	1.1	18.4	1.6
**Fruit (apple, pear, peach, grapes, bananas, tangerin)**	140	15.1%	83.5	114.9	1.1	0.3	43.0	2.4

**Milk and milk derivatives (cow's milk, mozzarella, parmesan, cheese)**	290	31.4%	314.7	232.06	21.23	22.15	20.78	0.0
**Meats, meat derivatives, fish**	120	13%	157.7	83.9	26.0	4.6	6.2	0.0
**Egg and derivatives**	30	3.2%	91.1	16.9	2.9	8.7	0.4	0.0
**Oil and fats (Extra virgin olive oil and butter)**	25	2.7%	210.65	1.4	0.1	23.3	0.2	0.0
**Peanuts**								
**Sugar and Honey**	10	1.1%	38.7	0.0	0.0	0.0	10.0	0.0
**Snacks**	30	3.2%	179.55	18.8	2.2	8.7	34.9	0.0
**Total daily food intake**	**925**		**1512.7**		**66.7**	**73.9**	**290.0**	**8.4**

* Average of max Quantity ingested per child per day

Supplementary Table 3. Amounts of SCFAs in fecal samples from African and European children

		µmol/g feces				
**ID_subject**	**Group**	**tot SCFAs**	**ACETIC**	**BUTANOIC**	**PROPANOIC**	**PENTANOIC**
2BF	BR	103.80	58.25	8.95	35.00	1.60
3BF	BR	88.35	51.80	15.95	16.30	4.30
4BF	BR	39.00	25.85	1.80	10.65	0.70
6BF	BR	47.40	29.80	5.50	10.90	1.20
7BF	BR	83.40	37.50	19.80	24.90	1.20
8BF	BR	205.70	129.90	24.50	49.60	1.70
9BF	BR	137.40	74.05	19.85	40.45	3.05
10BF	BR	42.15	26.30	3.95	11.20	0.70
11BF	BR	95.50	40.95	20.45	33.15	0.95
12BF	BR	57.15	29.00	7.25	19.55	1.35
13BF	BR	31.75	23.30	3.45	4.60	0.40
15BF	BR	33.40	15.55	5.00	12.35	0.50
16BF	BR	49.75	42.60	6.25	0.10	0.80
17BF	BR	164.95	65.20	18.40	79.95	1.40
**Mean**		**84.3**	**46.4**	**11.5**	**24.9**	**1.4**
SEM		14.27	7.87	2.10	5.71	0.28
1BT	BT	22.99	12.58	8.22	1.91	0.28
2BT	BT	43.18	19.47	5.86	17.62	0.23
3BT	BT	37.22	24.12	4.34	8.39	0.37
4BT	BT	39.89	20.03	8.95	9.08	1.83
5BT	BT	30.23	12.40	6.17	11.22	0.44
6BT	BT	44.44	22.16	7.07	14.52	0.70
7BT	BT	25.06	13.57	4.40	6.17	0.91
8BT	BT	35.76	18.80	8.99	6.44	1.53
**Mean**		**34.84**	**17.89**	**6.75**	**9.42**	**0.79**
SEM		2.84	1.59	0.66	1.76	0.21
1BC	BC	38.32	24.65	11.80	1.27	0.60
2BC	BC	41.22	24.69	5.95	10.05	0.54
3BC	BC	81.16	43.90	14.49	20.19	2.58
4BC	BC	51.44	36.82	11.55	2.64	0.42
5BC	BC	39.26	26.25	8.49	4.30	0.23
**Mean**		**50.28**	**31.26**	**10.46**	**7.69**	**0.88**
SEM		0.36	0.28	0.32	1.01	1.10
1EU	EU	33.8	21.9	3.5	8.2	0.2
2EU	EU	27.7	18.2	3.1	6	0.4
3EU	EU	15.25	10.41	0.42	3.83	0.59
5EU	EU	29.3	17.7	6	5.2	0.4
6EU	EU	36.1	25.6	2.8	7.5	0.2
8EU	EU	15.9	11.3	1.5	2.7	0.4
10EU	EU	23.1	18	1.1	3.6	0.4
11EU	EU	72.64	44.65	8.27	18.42	1.3
12EU	EU	64	44.9	2.15	15.6	1.35

13EU	EU	15.5	11	1.4	2.7	0.4
17EU	EU	21.2	15.5	1.5	3.9	0.3
18EU	EU	39.1	26.2	3.6	8.5	0.8
19EU	EU	19.9	17.2	0.7	1.6	0.4
20EU	EU	19.2	15	0.8	3	0.4
21EU	EU	19.4	15.5	0.7	2.8	0.4
**Mean**		**30.14**	**20.87**	**2.50**	**6.24**	**0.53**
SEM		4.47	2.78	0.57	1.26	0.09

Supplementary Table 4. P-values by Student T test derived by comparison of SCFAs levels in the four populations.

**p-value**	**ACETIC**	**BUTANOIC**	**PROPANOIC**	**PENTANOIC**	**tot SCFA**
BR vs EU	0.0020	0.0001	0.0014	0.0025	0.0005
BR vs BT	0.0070	0.0552	0.0296	0.0699	0.0090
BT vs EU	0.2330	0.0001	0.0771	0.1047	0.2390
BR vs BC	0.1400	0.3892	0.0504	0.1655	0.0938
EU vs BC	0.0338	0.00001	0.3125	0.1202	0.0193
BT vs BC	0.0018	0.0120	0.3150	0.4191	0.0276

Supplementary Table 5. P-values by Wilcoxon rank sum test derived by pairwise comparison of taxonomic abundances at (*A*) phylum, (*B*) family, and (*C*) genus level, among the four populations. P-values and p-values adjusted with FDR correction are reported.

(*A*)

	BR vs BT		BR vs BC		BR vs EU		BT vs BC		BT vs EU		BC vs EU	
Phylum	p	p.adj (FDR)	p	p.adj (FDR)	p	p.adj (FDR)	p	p.adj (FDR)	p	p.adj (FDR)	p	p.adj (FDR)
Euryarchaeota	0.8767	0.8767	0.5896	0.6633	0.3156	0.3472	0.5271	0.6134	0.2393	0.3291	NA	NA
Actinobacteria	0.0019	0.0232	0.0022	0.0124	0.0000	0.0001	0.0295	0.3248	0.0298	0.0665	0.4430	0.4984
Bacteroidetes	0.0506	0.1519	0.0055	0.0124	0.0000	0.0000	0.2844	0.5214	0.0302	0.0665	0.3488	0.4984
Elusimicrobia	0.4555	0.5368	0.5896	0.6633	0.3156	0.3472	NA	NA	NA	NA	NA	NA
Firmicutes	0.1518	0.3642	0.0055	0.0124	0.0001	0.0002	0.2222	0.5214	0.0638	0.1170	0.5663	0.5663
Fusobacteria	0.2864	0.4910	NA	NA	0.0550	0.1008	0.5271	0.6134	0.3828	0.4679	0.1981	0.3566
Lentisphaerae	0.2864	0.4910	NA	NA	0.2046	0.2814	0.5271	0.6134	1.0000	1.0000	0.4167	0.4984
Proteobacteria	0.4920	0.5368	0.4409	0.6614	0.5309	0.5309	0.1274	0.5214	0.8044	0.8849	0.0945	0.2836
Spirochaetes	0.4808	0.5368	0.0043	0.0124	0.0000	0.0000	0.2780	0.5214	0.0071	0.0389	0.1366	0.3074
Tenericutes	0.0504	0.1519	0.3333	0.6000	0.1310	0.2059	0.8171	0.8171	0.0019	0.0212	0.0241	0.1726
Unknown	0.3511	0.5266	0.9548	0.9548	0.0077	0.0169	0.5576	0.6134	0.0887	0.1393	0.0383	0.1726
Verrucomicrobia	0.0375	0.1519	NA	NA	NA	NA	0.1658	0.5214	0.0238	0.0665	NA	NA

(*B*)

	BR vs BT		BR vs BC		BR vs EU		BT vs BC		BT vs EU		BC vs EU	
Family	p	p.adj (FDR)	p	p.adj (FDR)	p	p.adj (FDR)	p	p.adj (FDR)	p	p.adj (FDR)	p	p.adj (FDR)
Bacteroidetes_ Prevotellaceae	0.033	0.154	0.002	0.014	0.000	0.000	0.047	0.258	0.001	0.005	0.833	0.952
Firmicutes_Ru minococcaceae	0.026	0.143	0.009	0.021	0.000	0.000	0.622	0.864	0.089	0.173	0.035	0.136
Firmicutes_Lac hnospiraceae	0.238	0.490	0.005	0.016	0.018	0.038	0.093	0.284	0.140	0.257	0.173	0.370
Bacteroidetes_ Bacteroidaceae	0.117	0.370	0.000	0.005	0.000	0.000	0.019	0.205	0.004	0.016	0.924	0.986
Unknown_Unk nown	0.351	0.576	0.955	0.986	0.008	0.018	0.558	0.864	0.089	0.173	0.038	0.136
Firmicutes_Un known	0.043	0.158	0.052	0.110	0.042	0.074	0.213	0.530	0.612	0.703	0.443	0.626
Actinobacteria _Bifidobacteria ceae	0.038	0.156	0.004	0.015	0.000	0.000	0.060	0.282	0.006	0.019	0.443	0.626
Bacteroidetes_ Porphyromona daceae	0.005	0.055	0.027	0.062	0.000	0.000	0.724	0.885	0.011	0.026	0.059	0.190
Bacteroidetes_ Rikenellaceae	0.132	0.370	0.000	0.005	0.000	0.000	0.029	0.239	0.001	0.006	0.016	0.113
Proteobacteria_ Enterobacteriac eae	0.614	0.751	0.774	0.854	0.159	0.219	1.000	1.000	0.560	0.703	0.457	0.626
Firmicutes_Un known.1	0.172	0.437	0.570	0.689	1.000	1.000	0.379	0.736	0.310	0.487	0.489	0.626
Bacteroidetes_ Unknown	0.310	0.558	0.001	0.007	0.000	0.000	0.011	0.179	0.000	0.005	0.285	0.480

Proteobacteria_Enterobacteriaceae.1	0.901	0.934	0.063	0.125	0.000	0.000	0.233	0.530	0.002	0.009	0.137	0.312
Firmicutes_Veillonellaceae	0.321	0.558	0.733	0.838	0.353	0.432	0.833	0.982	0.689	0.758	0.961	0.992
Firmicutes_Streptococcaceae	0.934	0.934	0.377	0.575	0.139	0.200	0.557	0.864	0.346	0.519	0.730	0.898
Spirochaetes_Spirochaetaceae	0.588	0.751	0.004	0.015	0.000	0.000	0.089	0.284	0.007	0.019	NA	NA
Firmicutes_Clostridiaceae.1	0.383	0.576	0.570	0.689	0.171	0.226	0.941	1.000	0.942	0.942	1.000	1.000
Proteobacteria_Sutterellaceae	0.004	0.055	0.000	0.005	0.000	0.000	0.089	0.284	0.000	0.005	0.004	0.067
Proteobacteria_Unknown	0.185	0.437	0.003	0.015	0.036	0.066	0.095	0.284	0.769	0.818	0.025	0.113
Bacteroidetes_Unknown.1	0.384	0.576	0.005	0.016	0.000	0.000	0.278	0.573	0.007	0.019	0.137	0.312
Proteobacteria_Unknown.1	0.218	0.479	1.000	1.000	0.047	0.078	0.524	0.864	0.618	0.703	0.240	0.452
Actinobacteria_Coriobacteriaceae	0.023	0.143	0.099	0.187	0.839	0.865	0.714	0.885	0.011	0.026	0.012	0.113
Firmicutes_Peptostreptococcaceae	0.934	0.934	0.252	0.424	0.430	0.489	0.137	0.376	0.579	0.703	0.359	0.575
Firmicutes_Erysipelotrichaceae	0.932	0.934	0.004	0.015	0.392	0.462	0.010	0.179	0.442	0.608	0.001	0.037
Proteobacteria_Desulfovibrionaceae	0.001	0.037	0.008	0.021	0.001	0.003	0.881	1.000	0.152	0.265	0.269	0.478
Firmicutes_Leuconostocaceae	0.485	0.667	0.304	0.486	0.010	0.023	0.633	0.864	0.033	0.072	0.129	0.312
Proteobacteria_Unknown.2	0.134	0.370	0.515	0.686	0.001	0.003	0.047	0.258	0.000	0.001	0.024	0.113
Firmicutes_Unknown.2	0.464	0.665	0.476	0.681	0.627	0.690	1.000	1.000	0.793	0.818	0.760	0.900
Firmicutes_Lactobacillaceae	0.841	0.934	0.882	0.940	0.828	0.865	0.655	0.864	0.542	0.703	0.900	0.986
Fusobacteria_Fusobacteriaceae	0.286	0.556	NA	NA	0.055	0.086	0.527	0.864	0.383	0.549	0.198	0.396
Tenericutes_Unknown	0.012	0.101	0.157	0.279	0.316	0.401	0.589	0.864	0.002	0.009	0.024	0.113
Proteobacteria_Campylobacteraceae	0.726	0.856	0.582	0.689	0.073	0.109	0.921	1.000	0.259	0.427	0.470	0.626
Firmicutes_Unknown.3	0.612	0.751	0.489	0.681	0.023	0.044	0.241	0.530	0.007	0.019	0.137	0.312

(C)

	BR vs BT		BR vs BC		BR vs EU		BT vs BC		BT vs EU		BC vs EU	
Genus	p	p.adj (FDR)	p	p.adj (FDR)	p	p.adj (FDR)	p	p.adj (FDR)	p	p.adj (FDR)	p	p.adj (FDR)
Prevotellaceae_Prevotella	0.033	0.325	0.003	0.019	0.000	0.000	0.047	0.311	0.000	0.003	0.318	0.537
Ruminococcaceae_Unknown	0.051	0.335	0.013	0.048	0.000	0.000	0.724	0.872	0.037	0.095	0.026	0.178

Lachnospiraceae_Unknown	0.310	0.609	0.019	0.061	0.119	0.187	0.093	0.335	0.426	0.621	0.046	0.217
Bacteroidaceae_Bacteroides	0.117	0.509	0.000	0.006	0.000	0.001	0.019	0.247	0.004	0.017	0.924	0.942
Lachnospiraceae_Lachnospiracea_incertae_sedis	0.563	0.742	0.019	0.061	0.007	0.020	0.127	0.422	0.076	0.163	0.775	0.837
Unknown_Unknown	0.351	0.640	0.955	0.973	0.008	0.020	0.558	0.804	0.089	0.184	0.038	0.207
Clostridiales_Unknown	0.043	0.325	0.052	0.133	0.042	0.078	0.213	0.513	0.612	0.794	0.443	0.661
Lachnospiraceae_Roseburia	0.129	0.509	0.052	0.133	0.016	0.036	0.187	0.513	0.385	0.593	0.566	0.738
Bifidobacteriaceae_Bifidobacterium	0.038	0.325	0.004	0.021	0.000	0.000	0.060	0.335	0.006	0.021	0.443	0.661
Rikenellaceae_Alistipes	0.132	0.509	0.000	0.006	0.000	0.000	0.029	0.307	0.001	0.007	0.016	0.178
Prevotellaceae_Unknown	0.442	0.664	0.002	0.017	0.000	0.000	0.010	0.192	0.000	0.002	0.172	0.432
Ruminococcaceae_Oscillibacter	0.082	0.484	0.191	0.313	0.032	0.063	0.622	0.804	0.414	0.621	0.657	0.789
Enterobacteriaceae_Escherichia	0.706	0.796	0.774	0.835	0.278	0.391	1.000	1.000	0.610	0.794	0.729	0.837
Firmicutes_Unknown	0.172	0.581	0.570	0.654	1.000	1.000	0.379	0.652	0.310	0.507	0.489	0.661
Lachnospiraceae_Dorea	0.395	0.650	0.377	0.507	0.020	0.043	0.187	0.513	0.261	0.441	0.005	0.133
Bacteroidales_Unknown	0.310	0.609	0.001	0.010	0.000	0.000	0.011	0.192	0.000	0.007	0.285	0.496
Succinivibrionaceae_Succinivibrio	0.901	0.934	0.063	0.154	0.000	0.001	0.233	0.513	0.002	0.010	0.137	0.369
Veillonellaceae_Dialister	0.231	0.581	0.174	0.300	0.353	0.432	0.603	0.804	0.798	0.898	0.455	0.661
Streptococcaceae_Streptococcus	0.934	0.934	0.364	0.507	0.139	0.213	0.557	0.804	0.346	0.549	0.775	0.837
Porphyromonadaceae_Barnesiella	0.001	0.075	0.008	0.030	0.000	0.001	0.941	0.998	0.054	0.128	0.068	0.282
Porphyromonadaceae_Parabacteroides	0.405	0.650	0.040	0.114	0.003	0.008	0.339	0.652	0.039	0.095	0.200	0.432
Spirochaetaceae_Treponema	0.588	0.742	0.004	0.021	0.000	0.000	0.089	0.335	0.007	0.021	NA	NA
Lachnospiraceae_Coprococcus	0.649	0.748	0.394	0.507	0.485	0.534	0.378	0.652	0.913	0.967	0.693	0.813
Ruminococcaceae_Butyricicoccus	0.230	0.581	0.069	0.163	0.433	0.496	0.607	0.804	0.032	0.086	0.026	0.178
Gammaproteobacteria_Unknown	0.185	0.581	0.003	0.019	0.036	0.068	0.095	0.335	0.769	0.898	0.025	0.178
Clostridiums	0.525	0.714	0.388	0.507	0.479	0.534	0.824	0.929	0.685	0.824	0.653	0.789
Ruminococcaceae_Clostridium	0.617	0.743	0.306	0.459	0.244	0.353	0.656	0.809	0.687	0.824	0.960	0.960
Porphyromonadaceae_Odoribacter	0.286	0.607	0.001	0.012	0.000	0.000	0.045	0.311	0.001	0.007	0.048	0.217
Bacteroidetes_Unknown_Unknown	0.384	0.650	0.005	0.023	0.000	0.000	0.278	0.567	0.007	0.021	0.137	0.369
Proteobacteria_Unknown_Unknown	0.218	0.581	1.000	1.000	0.047	0.084	0.524	0.804	0.618	0.794	0.240	0.462
Sutterellaceae_Sutterella	0.004	0.107	0.000	0.006	0.013	0.031	0.208	0.513	0.646	0.811	0.839	0.888
Coriobacteriaceae_Collinsella	0.013	0.172	0.363	0.507	0.307	0.404	0.769	0.886	0.006	0.021	0.136	0.369

Peptostreptococcaceae_Clostridium	0.934	0.934	0.252	0.389	0.430	0.496	0.137	0.426	0.579	0.794	0.359	0.588
Lachnospiraceae_Blautia	0.506	0.706	0.209	0.331	0.431	0.496	0.091	0.335	0.942	0.978	0.489	0.661
Sutterellaceae_Parasutterella	NA	NA	0.039	0.114	0.000	0.001	0.081	0.335	0.001	0.007	0.036	0.207
Sarcina	0.445	0.664	0.159	0.300	0.000	0.001	0.381	0.652	0.002	0.010	0.137	0.369
Enterobacteriaceae_Klebsiella	0.582	0.742	0.709	0.782	0.745	0.774	0.474	0.784	0.829	0.914	0.574	0.738
Desulfovibrionaceae_Bilophila	0.286	0.607	0.008	0.030	0.001	0.004	0.089	0.335	0.010	0.028	0.248	0.462
Erysipelotrichaceae_Unknown	0.456	0.664	0.000	0.006	0.316	0.404	0.001	0.060	NA	NA	0.000	0.004
Burkholderiales_Unknown	0.134	0.509	0.515	0.618	0.001	0.004	0.047	0.311	1.96E-05	1.06E-03	0.024	0.178
Leuconostocaceae_Weissella	0.215	0.581	0.170	0.300	0.010	0.026	0.766	0.886	2.59E-01	4.41E-01	0.470	0.661
Porphyromonadaceae_Unknown	0.898	0.934	0.115	0.259	0.001	0.004	0.242	0.513	7.07E-03	2.12E-02	0.137	0.369
Lachnospiraceae_Clostridiumb	0.189	0.581	0.129	0.279	0.179	0.267	0.554	0.804	1.00E+00	1.00E+00	0.652	0.789
Porphyromonadaceae_Butyricimonas	0.104	0.509	0.178	0.300	0.013	0.031	0.843	0.930	1.71E-01	3.19E-01	0.217	0.452
Lactobacillales_Unknown	0.464	0.664	0.476	0.598	0.627	0.677	1.000	1.000	7.93E-01	8.98E-01	0.760	0.837
Lachnospiraceae_Clostridiuma	0.362	0.640	0.157	0.300	0.000	0.001	0.591	0.804	2.85E-03	1.40E-02	0.178	0.432
Lactobacillaceae_Lactobacillus	0.841	0.910	0.882	0.916	0.828	0.843	0.655	0.809	5.42E-01	7.70E-01	0.900	0.935
Ruminococcaceae_Ruminococcus	0.252	0.581	0.836	0.885	0.114	0.184	0.381	0.652	9.10E-01	9.67E-01	0.232	0.462
Erysipelotrichaceae_Erysipelotrichaceae_incertae_sedis	0.362	0.640	0.555	0.651	0.316	0.404	1.000	1.000	7.57E-02	1.63E-01	0.137	0.369
Prevotellaceae_Paraprevotella	0.245	0.581	0.374	0.507	0.341	0.426	NA	NA	1.01E-01	1.95E-01	0.198	0.432
Mollicutes_Unknown	0.012	0.172	0.157	0.300	0.316	0.404	0.589	0.804	1.92E-03	1.04E-02	0.024	0.178
Fusobacteriaceae_Unknown	NA	NA	NA	NA	0.055	0.094	NA	NA	1.01E-01	1.95E-01	0.198	0.432
Erysipelotrichaceae_Turicibacter	0.630	0.743	0.168	0.300	0.641	0.678	0.209	0.513	1.00E+00	1.00E+00	0.261	0.469
Campylobacteraceae_Campylobacter	0.726	0.802	0.582	0.654	0.073	0.121	0.921	0.996	2.59E-01	4.41E-01	0.470	0.661
Bacilli_Unknown	0.612	0.743	0.489	0.600	0.023	0.047	0.241	0.513	7.03E-03	2.12E-02	0.137	0.369

Supplementary Table 6. Nearest Sequenced Taxon Index (NSTI) by PICRUSt. NSTI is used to evaluate the accuracy of alignment of the subset of 16S rRNA sequences to their nearest sequenced reference genome.

Sample	Weighted NSTI_value
BR10	0.10
BR11	0.10
BR12	0.10
BR13	0.10
BR15	0.10
BR17	0.10
BR2	0.09
BR6	0.11
BR7	0.09
BR8	0.10
BR9	0.12
**NSTI mean ± s.d.**	**0.10 ± 0.009**
BT1	0.04
BT2	0.10
BT3	0.11
BT4	0.16
BT5	0.09
BT6	0.09
BT7	0.14
BT8	0.11
**NSTI mean ± s.d.**	**0.11 ± 0.03**
BC1	0.08
BC2	0.07
BC3	0.05
BC4	0.08
BC5	0.09
**NSTI mean ± s.d.**	**0.07 ± 0.01**
EU1	0.06
EU10	0.09
EU11	0.04
EU12	0.05
EU13	0.07
EU17	0.08
EU18	0.06
EU19	0.06
EU20	0.09
EU21	0.07
EU5	0.07
EU6	0.06
EU8	0.09
**NSTI mean ± s.d.**	**0.07 ± 0.01**

Supplementary Figures

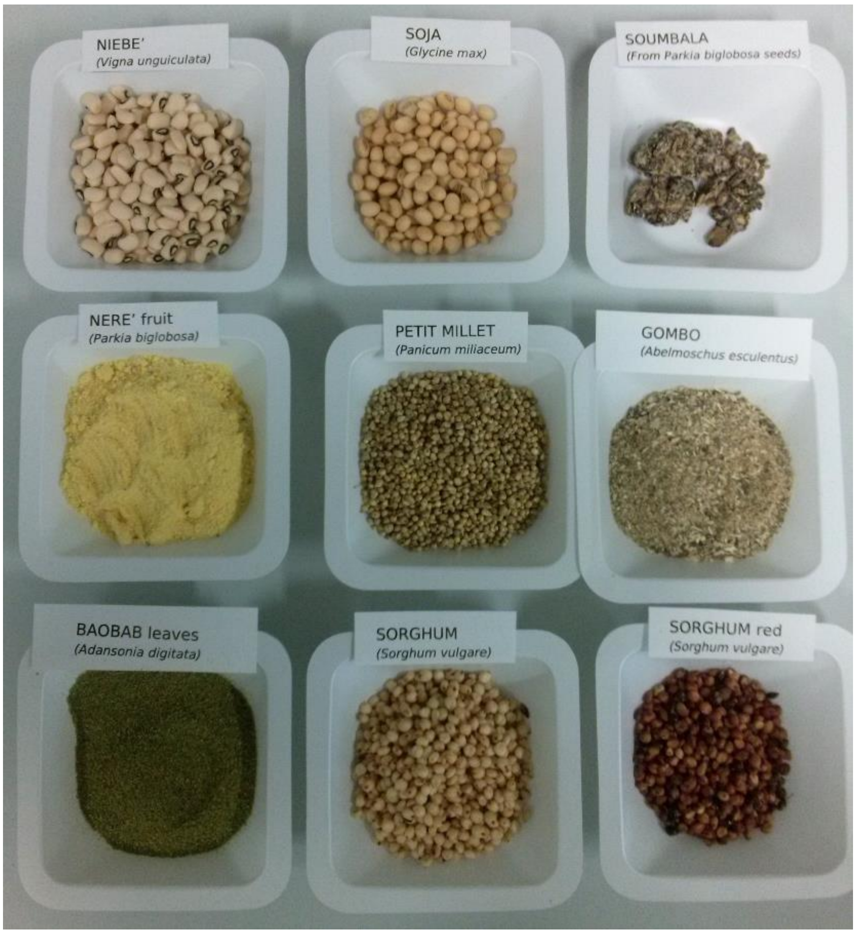

Supplementary Figure 1. Typical cereals, legumes and vegetables of traditional Burkina Faso diet. These foods are locally cultivated in rural Burkina Faso populations.

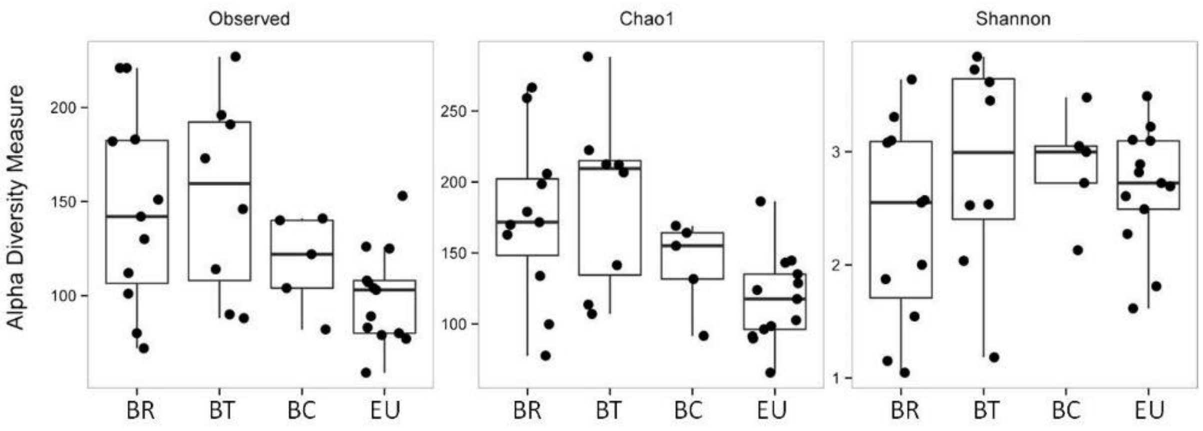

Supplementary Fig. 2. Box plots of alpha diversity in the three African populations compared to EU, based on number of observed OTUs, Chao 1 index and Shannon index.

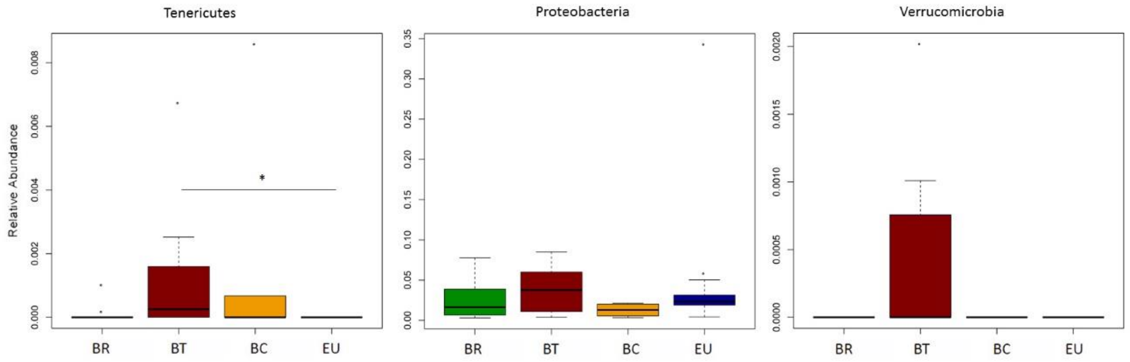

Supplementary Fig. 3. Box plot of relative abundances of minor components phyla of gut microbiota in African and European populations (Wilcoxon rank sum test; * p-value <0.05).

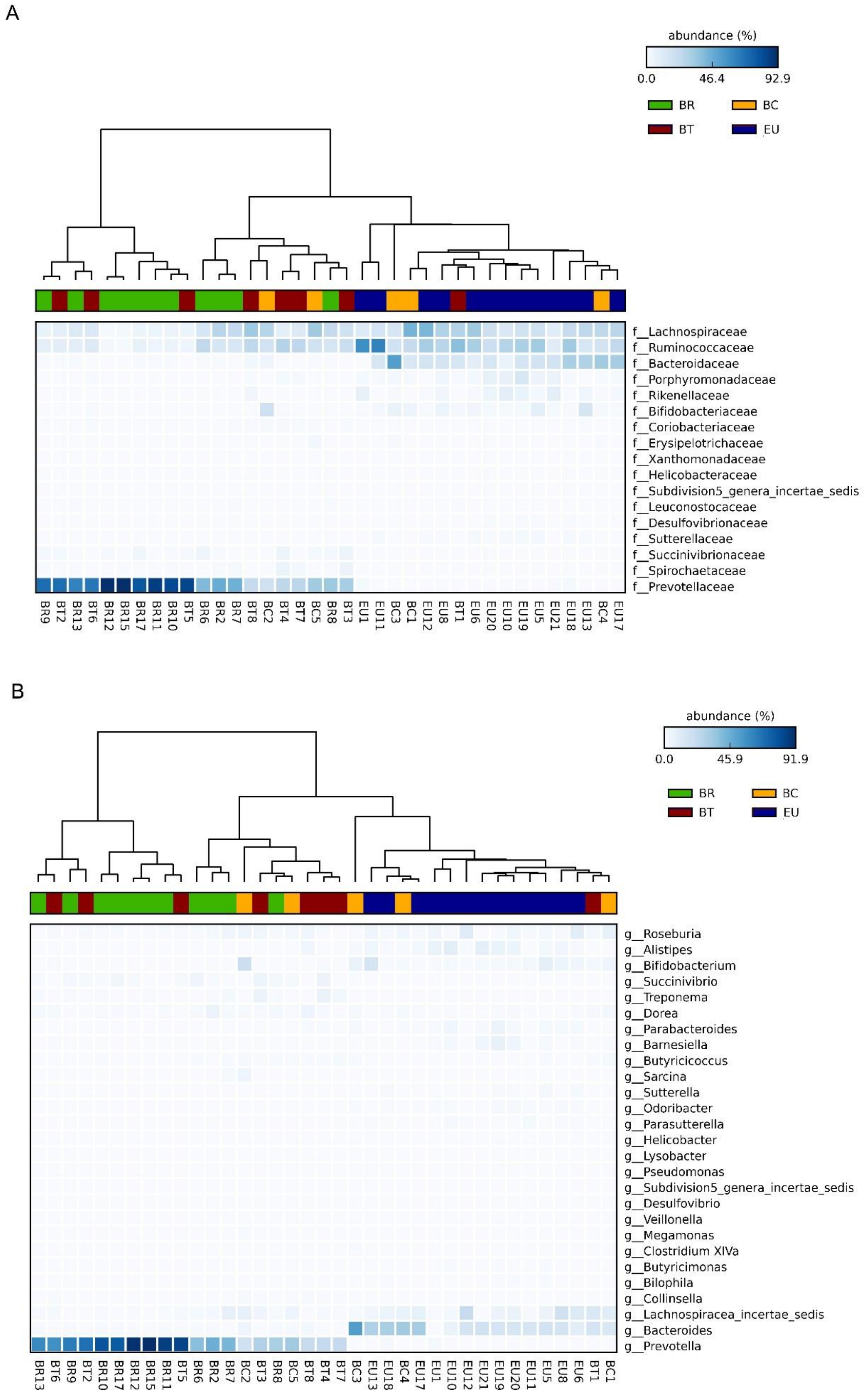

Supplementary Fig. 4. Clustering of African and European populations based on microbiota

composition**.** Heatmap plots report the sequence abundances (percentage; blue-scale squares) assigned at each (A) family and (B) genus in each fecal sample. Dendrograms, obtained with Average Neighbour UPGMA method, are used to cluster each fecal sample of the children populations (horizontal) based on (A) families and (B) genera abundances. Each sample, belonging to respective group, is represented by a different color: green=BR, brown=BT, yellow=BC and blue=EU.

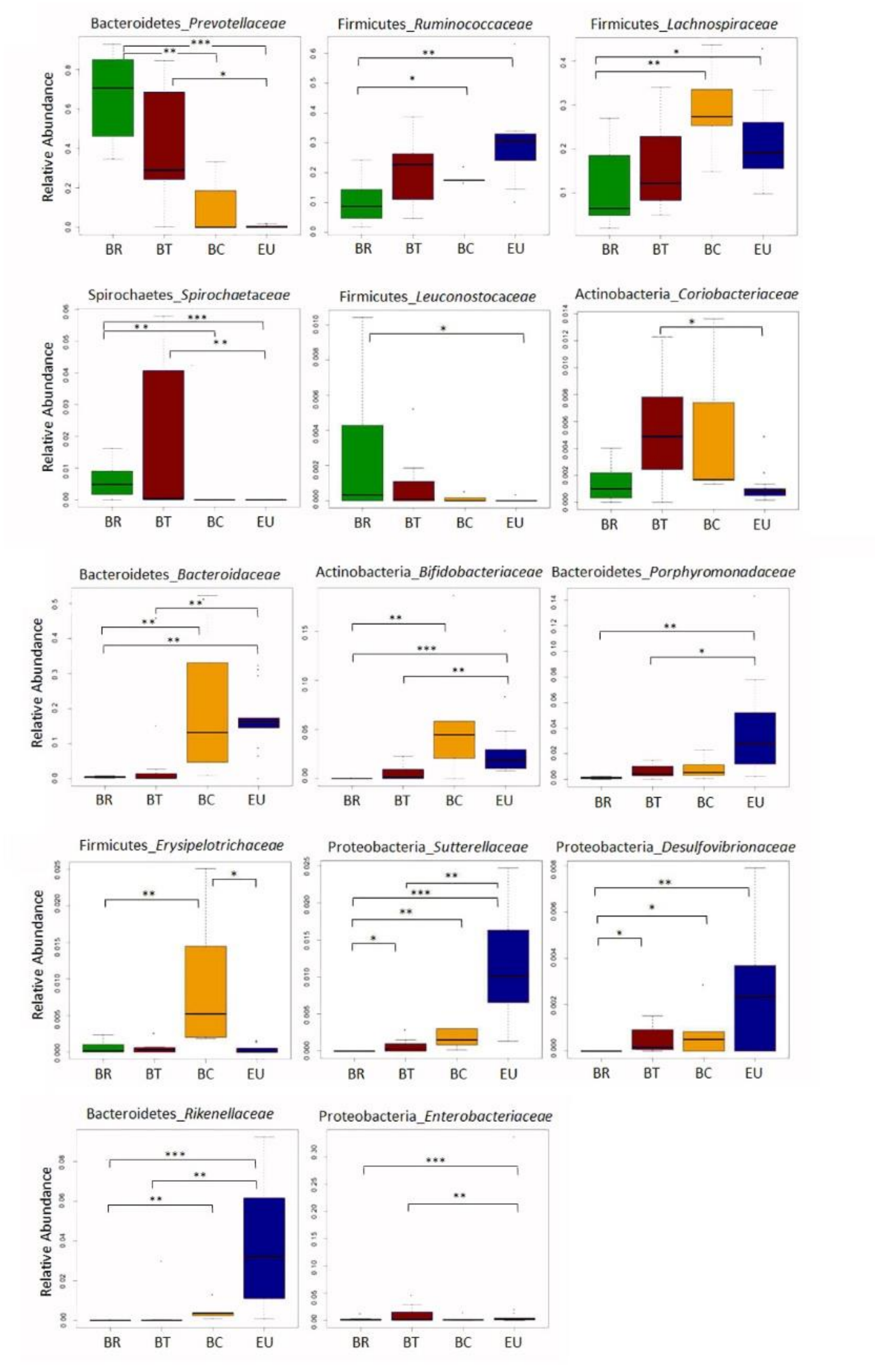

Supplementary Fig. 5 Box plot of relative abundances of the statistically significant different families in African and European populations (Wilcoxon pairwise test; *p-value <0.05, **p-value <0.01; ***p-value<0.001).

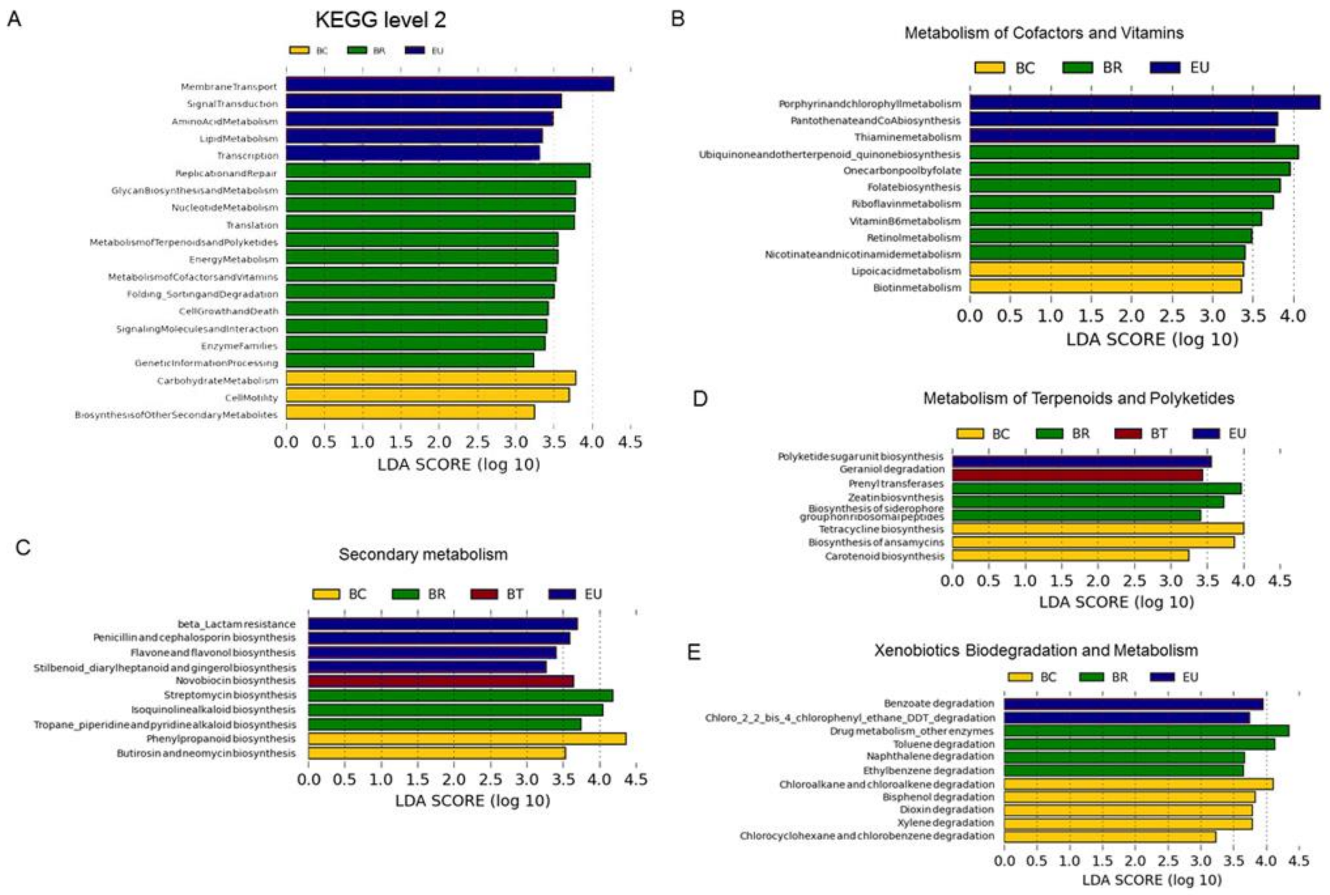

Supplementary Fig. 6 Differences in bacterial functional classes (KEGG categories). (A) Functional pathways significantly enriched in African and European populations based on PICRUSt prediction (KEGG categories level 2). (B-E) Secondary functional classes (KEGG categories level 3). LEfSe results indicate a sequentially significant ranking among populations (Alpha value=0.05 for the factorial Kruskal-Wallis test among classes). The threshold for the logarithmic LDA score was 2.0.
